# Sexual selection does not influence minisatellite mutation rate

**DOI:** 10.1186/1471-2148-9-5

**Published:** 2009-01-08

**Authors:** William Amos

**Affiliations:** 1 Department of Zoology, Cambridge University, Cambridge, UK

## Abstract

**Background:**

Moller and Cuervo report a significant trend between minisatellite mutation rate and the frequency of extra-pair copulations in birds. This is interpreted as evidence that the high rate of evolution demanded by sexual selection has itself selected for a higher mutation rate in species where selection is strongest. However, there are good a priori reasons for believing that their method of calculating minisatellite mutation rates will be highly error prone and a poor surrogate measure of the evolutionary rate of genes. I therefore attempted to replicate their results using both their data and an independent data set based on papers they failed to locate.

**Results:**

I find that Moller and Cuervo's data set contains numerous errors that act somewhat to strengthen their key regression. More importantly, data from uncited papers fail to replicate their reported trend and one species in particular, Vireo olivaceus, is apparently deliberately omitted, yet its inclusion removes significance from the original correlation. Over the small number of cases were comparisons can be made, mutation rate estimates do not differ between species but do vary significantly depending on the laboratory/operator.

**Conclusion:**

There appears to be no clear relationship between minisatellite mutation rate and EPC rate in birds. The previously reported trend can be attributed to data transcription errors and unfortunate data selection. My analysis highlights the importance of total methodological transparency when conducting meta-analyses.

## Introduction

Møller and Cuervo recently published an analysis of literature-based data purporting to show a positive relationship between the rate of extra pair paternity (EPP), a surrogate measure of sexual selection, and minisatellite mutation rate, a possible indicator of the genome-wide mutation rate [1]. Their intriguing idea is that sexually selected species need to evolve fast and have been selected for elevated mutation rates to facilitate this. If true, this would indeed be an important finding. However, the regression on which their main conclusion is based looks weak. Moreover, the estimation of minisatellite mutation rates, as described, embraces a wide range of sources of error that together make it unlikely to be accurate enough to detect even medium to strong trends.

Møller and Cuervo estimate minisatellite mutation rate using classical DNA fingerprint data from confirmed mother-father-offspring trios [1]. When the fingerprint profiles of a confirmed family are examined, bands are occasionally seen in an offspring that occur in neither parent [2]. Such bands are expected because the high mutation rate of minisatellites [3] combined with the number of bands surveyed in a DNA fingerprint means that mutated alleles are likely to be found with some regularity [2]. However, unassigned bands can also represent artefacts [4] such as refractory restriction enzyme cutting sites. Given that N novel fragments are observed in M verified mother-father-offspring trios, the mutation rate is then calculated as N/(M.B), where B is the average number of bands scored per individual. The result is an average mutation rate per band per generation. Unfortunately, minisatellite mutation rates vary over three or four orders of magnitude, from 0.15 per generation [5] or more down to 10^-3^, 10^-4 ^or less [6,7]. Truly hypervariable loci appear to be rare, at least in humans [6,8]. Consequently, most mutations will derive from one or a few of the most variable loci, and studies that score more bands will tend report lower mutation rates. With average band counts varying between 10 and over 50, this could easily create a 5-fold variation in perceived mutation rate that is unrelated to the actual mutability of the minisatellites themselves.

A second source of error is the variation in resolution between experiments and between studies. Published figures depicting gels, most of which will be the best-looking available to the author, vary in quality between studies from beautiful ladders of razor-sharp bands to series of indistinct blobs. This will impact both on the number of bands scored, and on the number of mutations identified. A key issue is that most minisatellite mutations involve small changes in fragment length, of the order of 50 bp or less [9-11], with an exponential fall-off in frequency with increasing numbers of repeat units that are lost or gained. For this reason, gel resolution will have a disproportionately big impact on measured mutation rate, with poor resolution gels picking up a small fraction of those resolved by the best gels. Although this effect tends to mitigate the impact of band number, from the perspective of measuring mutation rate it is an independent source of error that will further reduce the accuracy of this approach.

Given these problems with estimating mutation rate and the clear importance of this finding should it be correct, I decided to revisit Møller and Cuervo's original data to look for alternative explanations for the trend they report.

## Methods and results

With the exception of two PhD theses, I downloaded and re-extracted the data from all 92 references cited as source material in Møller and Cuervo's 'Supplementary material'. Hereafter, these papers are referred to by their original numbers, as they appear in table one of Møller and Cuervo's Supplementary material, prefixed by 'MC references'. When transcribing data that are largely embedded in text rather than table, it is easy to make errors. Consequently, I extracted all the required numbers twice, blind and cross-checked and corrected any discrepancies. In six papers (MC references 1, 24, 25, 46, 57 and 85), despite several careful readings of the paper, I failed to find data from which a mutation rate could be calculated. However, MC references 24 and 25 were redundant and I was able to substitute alternative papers by the same authors for MC references 1 (my reference [12]), 46 (my reference [13]) and 57 (my references [14,15]). For MC reference 85 I was unable to find any usable data and was forced to use their value. In some cases the meaning of the EPC rate value was unclear because the rate variously was the subject of experimental manipulation [16], varied with male age [17], varied between populations [18,19] or because the social organisation involved multi-male territories [20-22]. I ignored this variation.

### Data transcription accuracy

**Figure 1:**
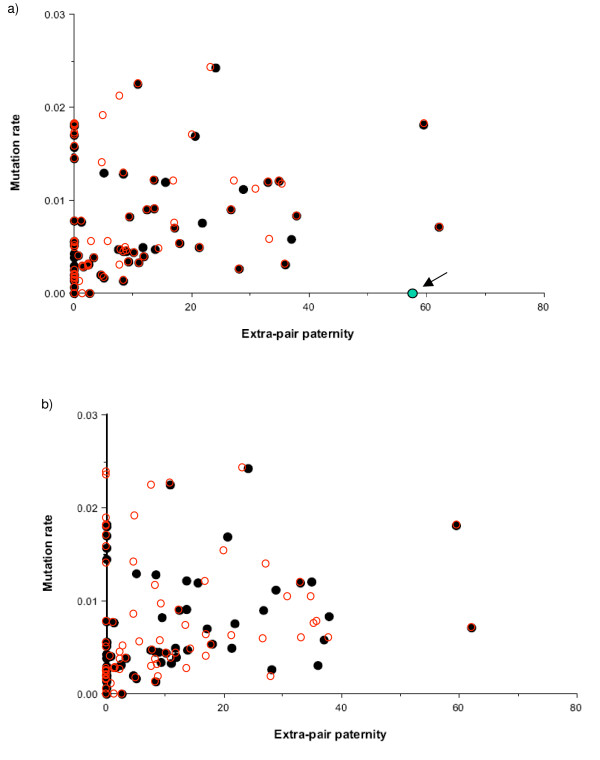
**Comparison between Figure 2 from Møller and Cuervo and the data it represents**. Figure 1a: Møller and Cuervo's Figure is copied directly from the PDF file (solid circles) and overlain with the same plot, based on the dataset given in Møller and Cuervo's Supplementary material table one (open circles). The arrowed data point represents *Vireo olivaceus*, a species for which data were available in one of the papers cited by Møller and Cuervo, but not used. This outlier greatly weakens the regression. Figure 1b: As for Figure 1a except this time the overlay data are based on values I extracted from the same source set listed in Møller and Cuervo's Supplementary material. Many discrepancies are apparent.

**Figure 2:**
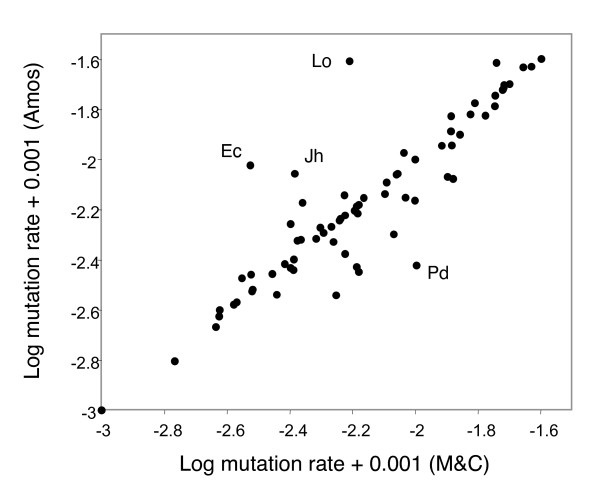
**Comparison between two sets of mutation rate estimates extracted from the same sources**. These data represent data extracted by myself and by Møller and Cuervo from the 92 references they site as sources. Large discrepancies are annotated with species name abbreviations: Lo = *Larus occidentalis*, Ec = *Embrezia calandra*, Jh = *Junco hyemalis *and Pd = *Passer domesticus*. Data for Passer domesticus were not available in the paper cited by Møller and Cuervo and were therefore taken from two alternative papers [14,15].

To examine the accuracy of data transcription I downloaded Møller and Cuervo's data from their Supplementary material table one and used it to replot their figure 2. There is noticeably poor agreement between the two graphs, illustrated by superimposing the two graphs (Figure 1a). To determine which set of values, if either, is correct, I next compared Møller and Cuervo's data with those I extracted from the same sources. Where data were not easily accessible (two PhD theses), not available (MC reference 85) or a complicated experimental design made it unclear how to calculate a meaningful value (MC reference 87) I used the same values as Møller and Cuervo. Superimposing my graph on Møller and Cuervo's figure 2 (Figure 1b) an even larger number of discrepancies are noticeable.

It is possible that Møller and Cuervo's figure 2 is based on an earlier, error prone version but that their Supplementary material is reasonably accurate. To test this I plotted my mutation rate values against those given in their Supplementary material (Figure 2). Some slight scatter is expected because about 10–15% of values had to be read from graphs and in some cases several alternative values could be calculated. For example, several papers give separate values of mean band counts for mothers, fathers and offspring separately. I chose to use the value for 'offspring', but one could argue either for taking the average of the two parents, or of all three values. However, appreciable numbers of points differ by more than can be accounted for in this way and there are also several large discrepancies. The reason for these discrepancies is unclear. For example, the lead author's own paper on swallows [23] states that 10 unattributable bands were found among 5271 scored (page 355, 'DNA fingerprinting'), giving a mutation rate of 10/5271 = 0.0019, yet the figure that appears in MC Supplementary material table one is 0.0026.

To explore the impact of the discrepancies I have identified on the key regression of mutation rate against EPC rate, I attempted to repeat exactly the regression performed in the original paper. For this, I followed their methods and transformed mutation rate data by taking the log of X + 0.001, while EPC rates were square root – arcsine transformed [1]. To control for phylogenetic non-independence, Møller and Cuervo use the program 'CONTINUOUS' [24,25]. I translated their phylogeny (their figure 1) into a Nexus format tree and used it in the program BayesTraits (available at ), which now incorporates the 'CONTINUOUS' program as an option. This program is used to calculate the likelihood of a null model, in which two traits are independent, with a model in which the two traits are correlated across, but not because of, the phylogeny. Using data from Møller and Cuervo's Supplementary material I was able, more or less, to replicate their reported p-value, calculated as -2 × (the difference in likelihoods between the two models) and interpreted as a Chi-squared value with 1 degree of freedom (my value, p = 0.01; their value, p = 0.013). Using the mutation rate data I extracted, the level of significance is reduced somewhat (Chi-squared = 5.69, 1 d.f., p = 0.017). Transcribed EPC rates show much greater consistency between my values and theirs, and impact little on the regression.

### Species selection

Møller and Cuervo do not say how they determined which species to use and which to omit, but one omission is particularly puzzling. Data from two different species occasionally appear in the same paper and in most cases both mutation rates are used (e.g. MC references 6 and 54). However, in one case data from two congeneric species, *Vireo solitarius *and *Vireo olivaceus*, occur alongside each other in the same paper [26], yet only data from *V. solitarius *are included in the regression. Both species are highly relevant because, despite being close relatives, one has very high rates of EPPs and the other very low. To examine the impact of this omission I added *V. olivaceus *to the main dataset (Figure 1a) and repeated the regression, again using BayesTraits and the same data transformations. Despite being only a single datapoint in almost 80 species, for Mølller and Cuervo's data, significance is reduced to borderline levels (Chi-squared = 3.955, 1 d.f., p = 0.0467). When the mutation rate values I extracted are used, the regression weakens further and becomes non-significant (Chi-squared = 3.335, 1 d.f., p = 0.068). Omission of this species is perhaps justifiable, given that the mutation rate is based on a single non-EPP chick, but if so, it needs to be explicitly justified.

**Figure 3:**
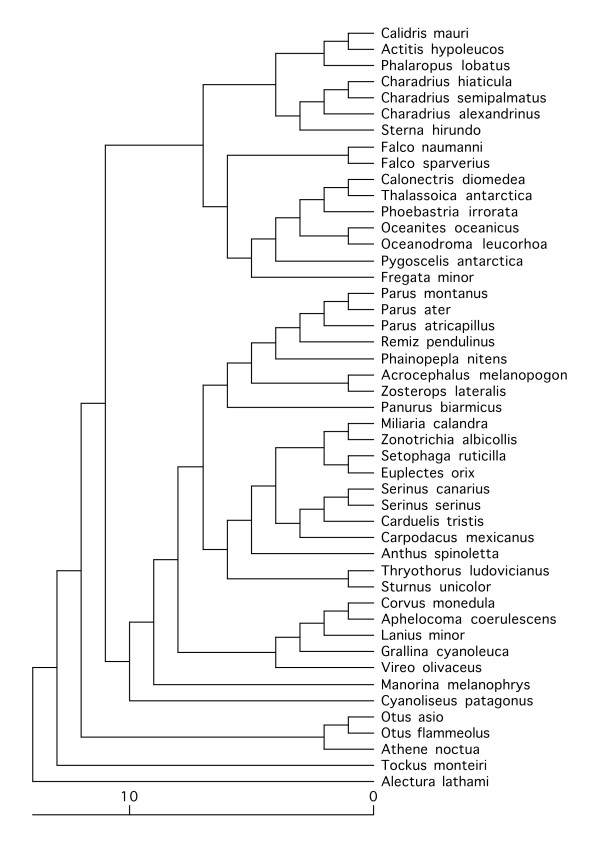
**Phylogeny of 47 species not studied by Møller and Cuervo where data were available for minisatellite mutation rates and extra-pair paternity**.

**Figure 4:**
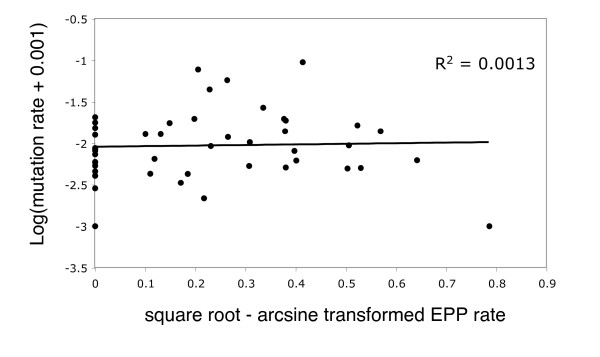
**Variation of minisatellite mutation rate with EPC rate in birds**. This graph attempts to repeat Figure 2 of Møller and Cuervo using a second, independent dataset of 47 observations. No relationship is seen and this is confirmed by a correlation analysis with phylogenetic correction conducted using the program BayesTraits.

In view of the borderline significance that results by adding one extra species and the number of papers I was finding that were not included by Møller and Cuervo, I decided to see whether the papers not used by Møller and Cuervo reveal a similar trend. To make my search repeatable and transparent (though not exhaustive) I used the terms 'fingerprint*' and 'extra-pair'/'extrapair' entered in the 'Web of Knowledge' ISI search engine . These terms returned a high proportion of all papers used by Møller and Cuervo, but also data for 46 further species not used in the original study (two papers that were not available electronically to Cambridge University members were ignored). I therefore constructed a phylogenetic tree, based on Møller and Cuervo's figure 1 plus a published phylogeny of the passerines (Figure 3) [27]. Since it is unclear how best to combine genetic distances across studies with vastly differing methodologies, I calculated a unit ultrametric tree (adjacent nodes separated by unit distance), with branch order determined by adding species to the reference phylogenies according to the position of their own or their most related genus. Using these data (see Figure 4, for full dataset see Additional File 1) and the same data transformations described above, BayesTraits revealed a non-significant correlation between minisatellite mutation rate and EPP frequency (Chi-squared = 0.32, 1 d.f., p = 0.57).

### Repeatability of minisatellite mutation rate estimates

Across the many source papers, mutation rate estimates are determined by many different operators working in different laboratories and using a wide range of protocols (including different restriction enzymes to cut the genomic DNA, different minisatellite probes to detect the banding profiles and different size ranges in which to score bands). For the Møller and Cuervo analysis to work, variation due to operator and methodology must be small relative to the actual variation in mutation rate between species. Møller and Cuervo present a number of tests to reassure the reader that this is so. They state that minisatellite mutation rate measurements show 'significant repeatability' among populations of the same species and for probes applied to the same population, and that mutation rate did not vary between different probes or enzymes. Their exact approach is not described beyond referring to Falconer and Mackay (see page 136) [28].

In practice, repeatability appears rather low. Thus, multiple measurements from the same species often vary by up to an order of magnitude or more (*Branta leucopsis*, range 0.0008 to 0.014; *Delichon urbica*, range 0.0015 to 0.0079; *Parus major*, range 0.00057 to 0.008; *Passer domesticus*, range 0.00059 to 0.0068; *Tachycineta bicolour*, range 0.0045 to 0.041; *Wilsonia citrina*, range 0.0014 to 0.0155), about the same range seen across all species (maximum value = 0.024, minimum value set for log transformation = 0.001). Moreover, there are very few data on which a valid test of repeatability can be conducted: there are only four cases of direct comparability between laboratories (i.e. studies by different laboratories using the same species, enzyme and probe: MC references 4 v 5, 79 v 80, 81 v 82 and 91 v 92) and only two direct comparisons between species (different species studied by the same operator, enzyme and probe: MC references 21 v 22 and 88 v 91). Three further papers report data for two species side-by-side, but in each case the species are closely related (MC references 6, 51 and 54), undermining the validity of any comparison.

To obtain adequate sample sizes for tests of repeatability it is necessary to assume that differences between probes and enzymes are small enough to be ignored (this is probably not valid, see below). Doing this, I find 11 instances where the same first author has studied at least two distantly related species, revealing significant differences between authors (ANOVA, F_[10,12] _= 4.34, p = 0.009), with author explaining 5.5% of the total variation. I also found nine species that were genotyped by different laboratories (*Agelaius phoeniceus, Branta leucopsis, Delichon urbica, Ficedula hypoleuca, Parus caerulescens, Parus major, Progne subis, Sturnus vulgaris and Tachycineta bicolour*) but mutation rates did not vary significantly among these species (ANOVA, F_[8,13] _= 2.015, p = 0.126). Thus inter-laboratory variation appears larger than differences between species.

Møller and Cuervo also claim that mutation rate estimates do not vary with enzyme or probe [1]. Again, the precise test used is not revealed, but, for example, it is stated that use of restriction enzyme Alu I does not differ from the use of other enzymes, the test being cited as "(F = 0.04, d.f. = 1,60, p = 0.83)". This implies a regression based on 62 paired mutation estimates for Alu I and one or more other enzymes. However, I can find only 23 studies that use Alu I and of these only two give separate mutation rates for Alu I and another enzyme (one of these states that the two rates are not independent because of extensive band overlap). For enzyme Hae III a similar test is reported with an identical number of degrees of freedom (1,60), even though many more studies, 52, use this enzyme. The only way I can see to reconcile the quoted degrees of freedom with the data available would be if a two-sample t-test (presented as an ANOVA) had been conducted to test the null hypothesis that average mutation rate for the two different enzymes does not differ between studies, but this would be (obviously) pointless because it fails to control for variation due to 'species', 'operator' or 'probe', all of which will conflate to increase the error variance.

## Discussion

Møller and Cuervo present an intriguing analysis purporting to show a link between sperm competition, as indicated by EPP rate, and minisatellite mutation rate [1]. Reanalysing the raw data I find numerous errors in the transcription of mutation rate data from the original paper. The outcome of the key regression depends critically on the omission of one highly influential data point. Including this species removes significance from the regression. Analysis of equivalent data extracted from a second, independent set of papers fails to reveal any trend. I also failed to replicate their claimed repeatability among species, instead finding that the most important source of variation is attributable to differences among laboratories/operators not to differences between species.

The idea of using putative mutations identified in parentage studies conducted using DNA fingerprinting as a surrogate measure of genome-wide mutation rate that can in turn be linked to ecological variables is ingenious. However, the data derive from many different laboratories, using different methods to study different species, and it is therefore vital to show that a significant proportion of the variation in estimated mutation rate is due to differences between species rather than between operators or methods. Møller and Cuervo claim to show this by reporting a series of tests of repeatability, but their quoted degrees of freedom are difficult to reconcile with the actual (tiny) number of valid comparisons that can be made. When I tried to conduct similar tests I was forced to ignore differences in methodology in order to find enough comparisons among species genotyped by the same laboratory and laboratories that had genotyped multiple species. In contrast to their reported 'significant repeatability' among populations of the same species, the only significant differences I find are, as expected, among operators.

In an attempt to replicate their repeatability analysis, I was forced to assume that the use of different probes and different enzymes makes little difference to the mutation rate estimate, but this assumption is probably false. Several studies report significant differences in band-sharing and/or band number between probes or enzymes tested on the same species (e.g. [29]). Band-sharing tends to reflect mutation rate (higher mutation rates drive higher diversity and hence lower band-sharing) while band number will influence the mutation rate calculation (see above). Consequently, significant variation in either of these within a species implies strongly that choice of probe/enzyme will influence the resulting mutation rate estimate. In my own work I have observed massive differences in band-sharing between difference probes used on the same species, implying equivalently large differences the underlying mutation rate (pers. obs.). Among the bird studies, some workers have used microsatellite probes such as (GATA)_n _rather than true minisatellite probes [30]. Here again a large difference in mutation rate is expected because maximum tetranucleotide repeat mutation rates are of the order of 10 fold lower than those of minisatellites. Again, one would expect probe choice to influence the mutation rate reported.

Putting aside the question of whether sufficient signal exists to be detected, I also failed to replicate Møller and Cuervo's regression of minisatellite mutation rate on EPC rate. There appear to be at least two important reasons for this. First, the data presented in Møller and Cuervo's Supplementary material, while apparently being those used in their calculations, fail to match either their own figure 2 or, more importantly, the data I extracted and double-checked from the same source papers. Although most of the differences are small, the net effect of these inaccuracies is to strengthen the reported relationship. Second, a much larger impact can be attributed to the species that were selected for inclusion. Møller and Cuervo's rationale for including or excluding data is opaque, and a parallel set of data culled from papers they omitted but that were published during the same time period reveals no tend whatsoever. Worryingly, the reported correlation depends critically on the omission of data for one particular species, *Vireo olivaceus*. Inclusion of this species causes Møller and Cuervo's regression to become borderline, whilst when added to the data I extracted from the same sources, the regression becomes no longer significant. Møller and Cuervo do not explain why this (or other) species were omitted, even though *V. olivaceus *could reasonably be excluded due to small sample size, but this nonetheless emphasises the need for complete transparency.

Some thought should also be given to possible artefacts associated with sample sizes and EPP assignment. For example, sample size of offspring varies among the studies from fewer than ten up to almost 1000. In the smallest studies, the chance of finding a low EPP rate together with no mutant bands could be much enhanced. Similarly, Møller and Cuervo assert that the combined use of band mismatches and band-sharing coefficients makes the identification of true offspring (and hence mutant bands) versus EPPs unambiguous. While this is true in many studies, there are some cases where no clear discontinuity is seen (e.g. [31,32]). Such studies will tend to report both high rates of EPPs and high mutation rates, creating outlier points with high leverage on the regression.

Finally, even ignoring the problems I have uncovered, two of the original premises appear fundamentally flawed. First, it is unclear why the mutation rate of the fastest evolving minisatellites should reflect the mutation rate of genes involved with sexual selection. Minisatellites evolve through a complicated process probably involving gene conversion like events that act either to delete repeat units or to copy blocks from one chromatid to the other [11]. This process has little if anything to do with the mechanisms responsible for the point mutations that are likely to be responsible for variation in genes associated with sexually selected traits. Consequently, even if selection is acting to increase the mutation rate of sexually selected genes, it seems unlikely to impact on minisatellite mutation rates; molecularly, the two process have too little in common. Second, there is the question of timescale. Point mutations typically occur at a rate of ~10^-9 ^per generation per site or ~10^-6 ^per generation per gene. If one assumes a generation length of three to five years for an 'average' bird, this implies changes occurring over a timescale of the order of five million years. In contrast, EPP rates can vary greatly between congeneric species and even among seasons and populations within a species [33], implying a timescale that might be faster by a factor of ten or more. Thus, while strong selection for evolutionary change might conceivably drive selection for increased genomic mutation rate, any resultant effect would probably affect larger clades rather than species, and is unlikely to correlate with the mechanistically unrelated minisatellite mutation rate.

## Conclusion

In conclusion, there are strong grounds for believing that minisatellite mutation rates estimated across diverse laboratories from occasional non-parental bands in DNA fingerprints will be highly variable and probably unrelated to point mutations, making them largely unsuitable as surrogate estimators of the genome-wide mutation rate. Reanalysing one study that attempts to use them in this way I find that the reported regression appears to be spurious and driven largely by data selection and data transcription errors. This analysis highlights the importance of transparency in meta-analyses, both in how data are selected and how the statistical tests were conducted. Accurate transcription of data is a seldom-checked but critical requisite!

## Response

A. P. Møller^1,2 ^and J. J. Cuervo^3^

^1 ^CNRS, UMR 7103, Laboratoire de Parasitologie Evolutive, Université Pierre et Marie Curie, Bât. A, 7ème étage, 7 quai St. Bernard, Case 237, F-75252 Paris Cedex 05, France;

^2 ^UPMC Paris 06, UMR 7103, Laboratoire de Parasitologie Evolutive, Université Pierre et Marie Curie, Bât. A, 7ème étage, 7 quai St. Bernard, Case 237, F-75252 Paris Cedex 05, France;

^3 ^Estación Experimental de Zonas Áridas, CSIC, Calle General Segura 1, E-04001 Almería, Spain

E-mail: amoller@snv.jussieu.fr

## Introduction

Our current understanding of the factors accounting for interspecific differences in mutation rates is at best poor [34]. We made a first attempt to address this lacuna by assembling a data base on mutation rates of minisatellites in different species of birds, produced as a result of a recent surge in studies investigating the evolution of extra-pair paternity [1]. The hypothesis tested was that sex differences in cell divisions could cause sex differences in mutation rates, and such sex differences should be more common in species with more intense sperm competition (see review of literature and justification for assumptions in [1]).

Amos [this article] suggested that we made numerous transcription errors from the original sources, omitted key species, and selectively included species that supported our hypothesis. We contest these assertions strongly and later explain in detail how they arose. We also provide a comprehensive data base with all our data, and we show that our previous conclusion remains even when increasing sample size from 77 to 132 species due to recently published data that have become available after we finished our first study, and when controlling for a number of novel, potentially confounding variables.

### How to estimate mutation rates

We were puzzled to learn that Amos [this article] had apparently not read or understood how we had estimated mutation rates, when we in fact described our procedures extensively [[1], p. 3, first column]. Given that Amos did not adopt the procedure that we used for estimating mutation rates, and given that he did not refute it on logical or other grounds, it is unsurprising that he finds extensive discrepancies between mutation rate estimates in the original papers and the rates that we reported. This does not justify claims about transcription errors or selective inclusion, but instead highlights the importance of reading papers before criticizing them.

Since Amos [this article] apparently cannot understand our explicit descriptions [[1], p. 3], we have no other choice than repeating ourselves. Whenever possible we estimated mutation rates directly from data in the original publications by extracting information on the distribution of novel bands that could not be attributed to extra-pair paternity directly from the text or figures. We also extracted information on the total number of bands scored and the number of individuals used for these analyses. However, as we explicitly stated in Møller & Cuervo [[1], p. 3], we should not include all individuals in such estimates because offspring caused by extra-pair parentage ((male attending a nest is not the father) and extra-pair maternity (female attending a nest is not the mother) and intraspecific nest parasitism (neither male nor female attending a nest are parents)) will bias mutation rate estimates. We explicitly provided two examples in Møller & Cuervo [[1], p. 3], but we restrict this repeated explanation to the first of our examples. This first example concerns the indigo bunting *Passerina cyanea *for which Westneat [35] analyzed extra-pair paternity of 63 young of which 22 were extra-pair offspring. He found that 28 nestlings had 0 novel bands, 10 had 1 novel band, and 3 had 2 novel bands, in total 16 novel bands in 41 nestlings. Westneat [35] scored on average 37.5 bands or 41 young × 37.5 bands/young = 1537.5 bands in total. The mutation rate is therefore 16/1537.5 = 0.010407. However, many papers also included extra-pair offspring in their reported estimates of mutation rates. If we had done so, we would have had 63 young × 37.5 bands/young = 2362.5 bands in total. That would have given a mutation rate estimate of 16/2362.5 = 0.006772, or a reduction in mutation rate estimate by 35%. We justified clearly why extra-pair offspring should be excluded from such estimates because extra-pair offspring have many novel bands (in the example with the indigo bunting on average 8.2 novel bands). We stated explicitly in Møller & Cuervo [[1], p. 3] and we re-iterate here that "The latter precaution was taken since a single or a few novel band(s) in an individual due to mutation cannot readily be distinguished among a large number of novel bands due to extra-pair paternity". Thus 'hidden' mutational bands in extra-pair offspring will cause a bias in mutation rate estimates, and, therefore, they have to be excluded. Here we now report the frequency distribution of novel bands, the mean number of bands scored per individual and the number of individuals used for estimating mutation rates in Additional file 2, allowing readers to assess all the data and confirm our estimates.

Finally, we note that some of the estimates of mutation rates reported in the original publications were at conflict with what could be estimated using the number of novel bands, the mean number of bands scored and the number of individuals, and also in these cases have we used our own estimate rather than what was reported in the paper.

### Transcription of data

Amos [this article] suggested that we made extensive transcription errors of mutation rates. We have already explained above why Amos [this article] did not find consistency between our estimates and what was reported in the original publications. He also explicitly stated that the mutation rate estimate that we reported from Gibbs et al. [36] could not be found in the paper. Opening the pdf file of Gibbs et al. [36] with Acrobat reader allows anybody to search for 'mutation' and find the estimate of 0.018 on p. 368!

We do not have access to Amos' complete data set, because he chose not to publish it, although we assume that this is the data in his Additional file 2 combined with the data that we reported as corrected by him, but adjusted for what he terms 'our transcription errors'. However, we cannot know if that is the case because this is never stated explicitly. We have copied the data listed in Additional file 2 in Amos [this article] and compared these values with what is reported in the original publications from which these data are claimed to have been extracted. We have found numerous errors. These range from the more mundane spelling errors in 9% of the species names (*Actitis hypoleucos*, *Carduelis tristis*, *Panurus biarmicus*, *Thryothorus ludovicianus*) to a difference in sample size of almost 1,000 for *Anthus spinoletta*. In addition, there are numerous errors in the frequency of extra-pair paternity, mutation rate, sample size and number of bands scored as reported in his Additional file 2. We report his data together with the data from the original publications in our Additional file 3 to allow readers to visualize these discrepancies. We suggest that someone who corrects others should be particularly careful not to make errors himself.

### Data selection criteria

Meta-analyses are strongly influenced by the data sets on which they are based, and it is always good scientific practice to report the data selection criteria adopted, but also to use multiple sources for accessing all available data [37-39]. We used all estimates of minisatellite mutation rates known to us in our first publication [1], and we would like to emphasize that one of us (APM) has kept an extensive list of all published studies of extra-pair paternity in birds since 1988, used for extensive analyses of the function of sperm competition [40,41]. We have never deliberately excluded any data, nor did we exclude *Vireo olivaceus*, contrary to what was suggested by Amos [this article]. Close scrutiny of the original publication [26] for this species revealed that although the estimate of extra-pair paternity was based on 19 nestlings, in fact only 8 nestlings had information on minisatellite bands for both parents. Among these 8 nestlings, no fewer than 7 were extra-pair offspring, leaving one single nestling for scoring mutational bands. Inclusion of an estimate of mutation based on a sample size of one should be clearly inadequate to anybody including Amos!

As a measure of the completeness of the entire data base we would like to emphasize that we have identified 15 publications that were not in our original data set nor in the data set reported by Amos [this article]. These papers are listed in our Additional file 2. As a second measure of completeness, we have kept a record of 48 manuscripts on extra-pair paternity that APM has refereed. Only one of these remains unpublished to date, suggesting that there is very little scope for any effects of publication bias in the analyses, contrary to what is commonly the case in meta-analyses [37-39].

### New analyses

Here we re-analyze the relationship between mutation rate and extra-pair paternity using the previously described procedures from Møller & Cuervo [1] and an extensive data set based on 132 species. In addition, we include five potentially confounding variables in the analyses. First, estimates based on large sample sizes will be more reliable than estimates based on small sample sizes, because the variance in estimates for small samples is greater than for large samples. Such patterns of decrease in sample variance with increasing sample size are ubiquitous in meta-analyses [37-39], and that is the main reason for including sample size as a confounding factor in the analyses. Thus it is not surprising that the variance also decreases with sample size in the present data for both mutation rates and extra-pair paternity. However, there is no reason to expect, as did Amos [this article], that estimates of extra-pair paternity and mutation rates will be inherently small and hence under-estimated at small sample sizes, because they will simply only be more variable. Hence, there is good reason to control for sampling effort. Second, the mean number of bands scored varied among studies, and a larger number of bands may suggest a greater level of precision and hence a greater probability of detecting novel bands. Third, as we have argued previously [1], and also in the present study, correction of mutation rate estimates for extra-pair paternity may reduce bias because novel bands due to mutation 'hidden' among bands due to extra-pair parentage will not contribute to estimates. Therefore, we included this variable as a factor (with studies where we extracted the information on mutation rate directly from the publication being scored as 0, and studies where we estimated mutation rate after exclusion of extra-pair parentage were scored as 1) in the analyses because we could not correct all mutation rate estimates due to missing values. Fourth, while we originally analyzed minisatellite mutations, Amos [this article] also included other molecular markers in the analyses. Hence, we included a factor that coded markers as minisatellites or other markers. Fifth, as we have already emphasized [1], molecular labs may differ in their procedures causing systematic differences in estimates of mutation rates among studies, and Amos [1] also suggested that there was a lab effect on estimates. Thus we included molecular lab as a factor in the analyses. Sixth, mutation rate estimates may depend on the number of bands scored if a larger number of bands imply a greater level of precision. Data for all these variables are provided in our Additional file 2 to allow readers to replicate our results and make further analyses. If more than one mutation rate and extra-pair paternity estimate was available for a species, we used mean estimates weighted by sample size for the analyses.

The best-fit model relating extra-pair paternity to mutation rate including no potentially confounding variable explained 5.1% of the total variance (Table 1A). A model weighted by the square-root of sample size minus three (the standard error, [25]) did not provide a better fit (Table 1B). The relationship between mutation rate and extra-pair paternity is shown in Fig 1 (see additional file 4). There was no significant additional effect of whether or not we extracted the mutation rate from the publication (*F *= 0.33, d.f. = 1,129, *P *= 0.57), whether the molecular marker was a minisatellite or not (*F *= 0.93, d.f. = 1,129, *P *= 0.34), identity of the molecular lab (*F *= 1.05, d.f. = 50,80, *P *= 0.41), or the mean number of bands scored (*F *= 3.95, d.f. = 1,120, *P *= 0.06). We constructed a phylogeny of all species (Fig 2, see additional file 5[45]) for analyses of the relationship between mutation rate and extra-pair paternity, while simultaneously considering similarity in phenotype among species due to common phylogenetic descent. This phylogenetic analysis provided similar conclusions to the analysis based on species-specific data, explaining 5.1% of the variance (*F *= 7.03, d.f. = 2,129, *P *= 0.0090). A phylogenetic analysis [25] weighted by the square-root of sample size minus three [42] did not provide a better fit to the data.

**Table 1:** Minisatellite mutation rates in different species of birds in relation to extra-pair paternity in (A) an analysis of species-specific unweighted data, and (B) an analysis of species-specific weighted data.

Variable	Sum of squares	d.f.	*F*	*P*	Slope (SE)
(A) Species-specific unweighted data					
Extra-pair paternity	1.077	1	7.03	0.0090	0.397 (0.150)
Error	19.920	1			
(B) Species-specific weighted data					
Extra-pair paternity	9.242	1	6.87	0.0098	0.408 (0.156)
Error	174.815	1			

Mutation rate was log_10_-transformed and a constant of 0.001 was added before transformation, extra-pair paternity was square-root arcsine-transformed.

### The relationship is conservative

The literature on mutation rates, and in particular the literature on mutations in minisatellites, is replete with comments on the difficulty of quantifying these, and Amos [this article] cites a number of these references. We are the first to acknowledge these difficulties. However, we deliberately attempted to quantify the influence of any of these sources of error by calculating repeatabilities, using standard procedures from the quantitative genetics literature based on one-way analysis of variance [28]. In this way Møller & Cuervo [1] could show that despite large heterogeneity in estimates, there were still significant repeatability in mutation rate estimates and estimates of extra-pair paternity. We could also show that restriction enzyme as a factor (code 1 for a given study using an enzyme and 0 for all other studies) did not explain variation in mutation rate estimates [1]. We showed that the minimum size of fragments scored did not explain mutation rate estimates [1]. Finally, we showed that there was no significant effect of molecular lab [1], and there was no temporal improvement in techniques as shown by no significant effect of year of publication [1]. The absence of significant effects still holds in the currently much larger data set.

The main finding of our study is that mutation rates and extra-pair paternity are significantly positively related, and this relationship accounts for 5.1% of the variance in an analysis of species-specific data and phylogenetically adjusted data, which equals a small to intermediate effect size (sensu Cohen [43], explaining 1% to 9% of the variance). We note that the effect size reported here is of the same magnitude that we originally reported (7.8%, [1]). This effect size is also very close to the average effect size in all meta-analyses in biology (around 5–7% of the variance explained [44]). Amos [this article] emphasized all the difficulties in estimating mutation rates, and that any relationship will be conservative. We can only concur that the many sources of noise in the data will render any biological signal much weaker than the true underlying signal. Hence, we consider the effect size of 5.1% to be an underestimate.

## Conclusion

In conclusion, we have corroborated our previous conclusion [1] that mutation rates increase with extra-pair paternity in birds, with an effect size of small to intermediate magnitude. This conclusion is robust to inclusion of a number of potentially confounding variables and to statistical control for similarity in phenotype among species due to common phylogenetic descent.

## Authors' contributions

APM wrote the paper.

APM and JJC extracted the data and finalized the paper.

## Supplementary Material

Additional file 1 **Full dataset for figure **4. Additional species with mutation rate (μ) and EPC rate (%) data. X = number of mutant bands, N = number of offspring analysed, Bands = number of bands per individual. In some cases a mutation rate is given without the data needed to calculate it: here columns X, N and Bands are left blank. Mutation rate is calculated as X/(N × Bands). Click here for file

Additional file 2 **Response Supplementary table 1**. Information on species, research laboratory, number of individuals with 0, 1, 2, 3, 4, 5 or 6 novel bands due to mutations, mean number of bands scored (always from nestlings if there were data for both adults and nestlings), number of offspring used for mutation estimation, total number of offspring, estimated mutation rate, mutation rate as reported in the publication, extra-pair paternity (%; if there was information based on more than one probe, then the estimate based on the largest sample size), genetic marker (only that/those used for estimating mutation rates), whether the molecular marker was a minisatellite, whether the study was included by Amos [1], and reference. References are listed in Møller & Cuervo [2] and Amos [1], and if not included there, the references are listed below. Click here for file

Additional file 3 **Supplementary Table 2.** Comparative information on extra-pair paternity, mutation rate, sample size and mean number of bands scored as reported by Amos [1] and according to the publications. Click here for file

Additional file 4 **Response figure **1. Positive relationship between mutation rate and extra-pair paternity (% extra-pair young) in different species of birds. Mutation rate was log_10_-transformed with a constant of 0.001 being added to avoid values of zero. The line is the linear regression line. Click here for file

Additional file 5 **Response figure **2. Phylogenetic relationships between the species of birds included in the analyses. Sources are listed in Møller & Cuervo [2], but now also include Hackett et al. [45]. Click here for file

## References

[B1] Møller AP, Cuervo JJ (2003). Sexual selection, germline mutation rate and sperm competition. BMC Evol Biol.

[B2] Burke T (1989). DNA fingerprinting and other methods for the study of mating success. Trends in Ecology and Evolution.

[B3] Jeffreys AJ, Wilson V, Thein SL (1985). Hypervariable 'minisatellite' regions in human DNA. Nature, London.

[B4] Fleischer RC, Tarr CL, Pratt TK (1994). Genetic structure and mating system in the palila, an endangered Hawaiian honeycreeper, assessed by DNA fingerprinting. Molec Ecol.

[B5] Buard J, Bourdet A, Yardley J, Dubrova Y, Jeffreys AJ (1998). Influences of array size and homogeneity on minisatellite mutation. EMBO J.

[B6] Denoeud F, Vergnaud G, Benson G (2003). Predicting human minisatellite polymorphism. Genome Res.

[B7] Langdon JA, Armour JAL (2003). Evolution and population genetics of the H-ras minisatellite and cancer predisposition. Hum Mol Genet.

[B8] Flint J, Bond J, Rees DC, Boyce AJ, Roberts-Thompson JM, Excoffier L, Clegg JB, Beaumont MA, Nichols RA, Harding RM (1999). Minisatellite mutation processes reduce Fst estimates. Hum Genet.

[B9] May CA, Jeffreys AJ, Armour JAL (1996). Mutation rate heterogeneity and the generation of allele diversity at the human minisatellite MS205 (D16S309). Hum Mol Genet.

[B10] Monckton DG, Neumann R, Guram T, Fretwell N, Tamaki K, MacLeod A, Jeffreys AJ (1994). Minisatellite mutation rate variation associated with a flanking DNA sequence polymorphism. Nature Genet.

[B11] Jeffreys AJ, Tamaki K, MacLeod A, Monckton DG, Neil DL, Armour JAL (1994). Complex gene conversion events in germline mutation at human minisatellites. Nature Genetics.

[B12] Hasselquist D, Bensch S, von Schantz T (1995). Low frequency of extrapair paternity in the polygynous great reed warbler, *Acrocephalus arundinaceus*. Behav Ecol.

[B13] Mulder RA, Dunn PO, Cockburn A, Lazenby-Cohen KA, Howell MJ (1994). Helpers liberate female fairy-wrens from constraints on extra-pair mate choice. Proc Roy Soc Lond B.

[B14] Wetton JH, Parkin DT, Carter RE (1992). The use of genetic markers for parentage analysis in *Passer domesticus *(house sparrows). Heredity.

[B15] Václav R, Hoi H, Blomqvist D (2003). Food supplementation affects extrapair paternity in house sparrows (*Passer domesticus*). Behav Ecol.

[B16] Raouf SA, Parker PG, Ketterson E, Nolan V, Ziegenfus C (1997). Testosterone affects reproductive success by influencing extra-pair fertilizations in male dark-eyed juncoes (Aves: *Junco hyemalis*). Proc Roy Soc Lond B.

[B17] Morton ES, Forman L, Braun M (1990). Extrapair fertilizations and the evolution of colonial breeding in purple martins. Auk.

[B18] Lifjeld JT, Dunn PO, Robertson RJ, Boag PT (1993). Extra-pair paternity in monogamous tree swallows. Anim Behav.

[B19] Barber CA, Robertson RJ, Boag PT (1996). The high frequency of extra-pair paternity in tree swallows is not an artefact of nestboxes. Behav Ecol Sociobiol.

[B20] Kempenaers B, Verheyen GR, Broeck M Van den, Burke T, Van Broeckhoven C, Dhondt AA (1992). Extra-pair paternity results from female preference for high-quality males in the blue tit. Nature.

[B21] Hartley IR, Davies NB, Hatchwell BJ, Desrochers A, Nebel D, Burke T (1995). The polyandrous mating system of the alpine accentor, *Prunella collaris*. II. Maultiple paternity and parental effort. Anim Behav.

[B22] Lundy KJ, Parker PG, Zahavi A (1998). Reproduction by subordinates in cooperatively breeding Arabian babblers is uncommon but predictable. Behav Ecol Sociobiol.

[B23] Møller AP, Tegelström H (1997). Extra-pair paternity and tail ornamentation in the barn swallow *Hirundo rustica*. Behav Ecol Sociobiol.

[B24] Pagel M (1994). Detecting correlated evolution on phylogenies: a general method for the comparative analysis of discrete characters. Proc Roy Soc Lond B.

[B25] Pagel M (1999). Inferring the historical patterns of biological evolution. Nature.

[B26] Morton ES, Stutchbury BJM, Howlett JS, Piper WH (1998). Genetic monogamy in blue-headed vireos and a comparison with a sympatric vireo with extrapair paternity. Behav Ecol.

[B27] Barker FK, Barrowclough GF, Groth JG (2001). Aphylogenetic hypothesis for passerine birds: taxonomic and biogeographic implications of an analysis of nuclear DNA sequence data. Proc Roy Soc Lond B.

[B28] Falconer DS, Mackay TFC (1996). Introduction to quantitative genetics.

[B29] Conrad KF, Robertson RJ, Boag PT (1998). Frequency of extrapair young increases in second broods of eastern phoebes. Auk.

[B30] Chuang HC, Webster MS, Holmes RT (1999). Extrapair paternity and local synchrony in the black-throated blue warbler. Auk.

[B31] Freeman-Gallant CR (1997). Extra-pair paternity in monogamous and ploygynous savannah sparrows, *Passerculus sandwichensis*. Anim Behav.

[B32] Martín-Vivaldi M, Martínez JG, Palomino JJ, Soler M (2002). Extrapair paternity in the hoopoe *Upupa epops*: an exploration of the influence of interactions between breeding pairs, non-pair males and strophe length. Ibis.

[B33] Langefors Å, Hasselquist D, von Schantz T (1998). Extra-pair fertilizations in the sedge warbler. J Avian Biol.

[B34] Baer CF, Miyamoto MM, Denevr DR (2007). Mutation rate variation in multicellular eukaryotes: causes and consequences. Nature Rev Genet.

[B35] Westneat DF (1990). Genetic parentage in the indigo bunting: a study using DNA fingerprinting. Behav Ecol Sociobiol.

[B36] Gibbs HL, Goldizen AW, Bullough C, Goldizen AR (1996). Parentage analysis of multi-male social groups of Tasmanian native hens (*Tribonyx mortieri*): genetic evidence for monogamy and polyandry. Behav Ecol Sociobiol.

[B37] Hedges LV, Olkin I (1985). Statistical methods for meta-analysis.

[B38] Rosenthal R (1991). Meta-analytic procedures for social research.

[B39] Møller AP, Jennions MD (2004). Testing and adjusting for publication bias. Trends Ecol Evol.

[B40] Birkhead TR, Møller AP (1992). Sperm competition in birds.

[B41] Birkhead TR, Møller AP, eds (1998). Sperm competition and sexual selection.

[B42] Møller AP, Nielsen JT (2007). Malaria and risk of predation: A comparative study of birds. Ecology.

[B43] Cohen J Statistical power analysis for the behavioral sciences.

[B44] Møller AP, Jennions MD (2002). How much variance can be explained by ecologists and evolutionary biologists?. Oecologia.

[B45] Hackett SJ, Kimball RT, Reddy S, Bowie RCK, Braun EL, Braun MJ, Chojnowski JL, Cox WA, Han K-L, Harshman J, Huddleton CJ, Marks BD, Miglia KJ, Moore WA, Sheldon FH, Steadman DW, Witt CC, Yuri T (2008). A phylogenomic study of birds reveals their evolutionary history. Science.

