# Supplementary material for: Sexual selection does not influence minisatellite mutation rate
Source: BMC Evol Biol. 2009 Jan 8;9:5. doi: 10.1186/1471-2148-9-5 (PMC2636768; doi:10.1186/1471-2148-9-5)
Supplement: Additional file 1 — Full dataset for figure 4. Additional species with mutation rate (μ) and EPC rate (%) data. X = number of mutant bands, N = number of offspring analysed, Bands = number of bands per individual. In some cases a mutation rate is given without the data needed to calculate it: here columns X, N and Bands are left blank. Mutation rate is calculated as X/(N × Bands). [file 1471-2148-9-5-S1.doc]

**Additional file 1:** Additional species with mutation rate (µ) and EPC rate (%) data. X = number of mutant bands, N = number of offspring analysed, Bands = number of bands per individual. In some cases a mutation rate is given without the data needed to calculate it: here columns X,N and Bands are left blank. Mutation rate is calculated as X / (N x Bands).

| **species** | **common name** | **EPC** | **µ** | **X** | **N** | **Bands** | **Ref** |
| --- | --- | --- | --- | --- | --- | --- | --- |
| *Acrocephalus melanopogon* | Moustached wrbler | 27 | 0.0155 |  |  |  | [1] |
| *Actidis hypoleucos* | Common sandpiper | 15.7 | 0.0071 | 19 | 57 | 47.1 | [2] |
| *Alectura lathami* | Australian brush turkey | 27.7 | 0.0040 | 5 | 47 | 26.3 | [3] |
| *Anthus spinoletta* | Alpine water pipit | 5.2 | 0.0436 | 151 | 71 | 48.8 | [4] |
| *Aphelocoma coerulescens* | Florida scrub jay | 1.4 | 0.0055 | 31 | 139 | 40.5 | [5] |
| *Athene noctua* | Little owl | 0 | 0.0036 | 4 | 53 | 20.95 | [6] |
| *Calidris mauri* | Western sandpiper | 7 | 0.0110 | 20 | 57 | 32 | [7] |
| *Calonectris diomedia* | Cory's shearwater | 0 | 0.0077 | 7 | 34 | 26.7 | [8] |
| *Carduilis tristis* | American goldfinch | 14.3 | 0.0130 | 14 | 54 | 20 | [9] |
| *Carpodacus mexicanus* | House finch | 14.4 | 0.0178 | 2 | 5 | 22.5 | [10] |
| *Charadrius alexandrinus* | Kentish plover | 3.9 | 0.0188 | 57 | 168 | 18 | [11] |
| *Charadrius hiacula* | Ringed plover | 0 | 0.0168 | 15 | 50 | 17.9 | [12] |
| *Charadrius semipalmatus* | Semipalmated plover | 4.7 | 0.0012 | 2 | 62 | 27.3 | [13] |
| *Corvus monedula* | Jackdaw | 0 | 0.0044 |  |  |  | [14] |
| *Cyanoliseus patagonus* | Burrowing parrot | 1.2 | 0.0033 | 14 | 165 | 25.6 | [15] |
| *Euplecter orix* | Red bishop | 14.1 | 0.0188 | 28 | 145 | 10.26 | [16] |
| *Falco naumani* | Lesser kestrel | 3.4 | 0.0033 | 3 | 84 | 10.9 | [17] |
| *Falco sparverius* | American kestrel | 11.2 | 0.0259 | 25 | 79 | 12.2 | [18] |
| *Fregate minor* | Frigate bird | 2.2 | 0.0165 | 21 | 90 | 14.1 | [19] |
| *Grallina cyanoleuca* | Australian magpie-lark | 2.9 | 0.0023 | 5 | 100 | 21.3 | [20] |
| *Lanius minor* | Grey shrike | 0 | 0.0049 | 14 | 136 | 21 | [21] |
| *Manorina melanophrys* | Bell miner | 4.2 | 0.0768 | 56 | 24 | 30.4 | [22] |
| *Miliaria calandra* | Corn bunting | 5.3 | 0.0083 | 4 | 36 | 13.4 | [23] |
| *Oceanites oceanicus* | Wilson's storm petrel | 0 | 0.0050 | 24 | 126 | 38.1 | [24] |
| *Oceanodroma leucorhoa* | Leach's storm petrel | 0 | 0.0078 | 7 | 42 | 21.4 | [25] |
| *Otus asio* | Eastern sceech owl | 0 | 0.0072 | 28 | 76 | 51 | [26] |
| *Otus flammeolus* | Flammulated owl | 0 | 0.0031 | 5 | 37 | 44 | [27] |
| *Panurus piarmicus* | Bearded tit | 14.4 | 0.0041 | 20 | 187 | 26 | [28] |
| *Parus ater* | Coal tit | 25.3 | 0.0085 | 14 | 118 | 14.03 | [29] |
| *Parus atricapillus* | Black-capped chickadee | 17 | 0.0941 | 101 | 53 | 20.26 | [30] |
| *Parus montanus* | Willow tit | 1 | 0.0120 | 26 | 111 | 19.5 | [31] |
| *Phainopepla nitens* | Phainopepla | 0 | 0.0063 | 6 | 34 | 27.8 | [32] |
| *Phoebastria irrorata* | Waved albatross | 25 | 0.0040 |  |  |  | [33] |
| *Phalaropus lobatus* | red-necked phalarope | 1.7 | 0.0120 |  |  |  | [34] |
| *Pygoscelis antarctica* | Chinstrap penguin | 0 | 0.0074 | 6 | 76 | 10.6 | [35] |
| *Remiz pendulinus* | Penduline tit | 6.9 | 0.0568 | 68 | 105 | 11.4 | [36] |
| *Serinus canaria* | Canary | 0 | 0.0019 |  |  |  | [37] |
| *Serinus serinus* | Serin | 9.4 | 0.0044 | 14 | 153 | 21 | [38] |
| *Setophaga ruticilla* | American redstart | 40 | 0.0052 | 5 | 62 | 15.4 | [39] |
| *Sterna hirundo* | Common tern | 0 | 0.0118 | 7 | 29 | 20.5 | [40] |
| *Sturnus unicolor* | Spotless starling | 16 | 0.0052 | 24 | 270 | 17.06 | [41] |
| *Thalassoica antarctica* | Antarctic petrels | 9.5 | 0.0094 | 11 | 38 | 30.9 | [42] |
| *Thyothorus ludovicianus* | Carolina wrens | 0 | 0.0197 | 22 | 84 | 13.3 | [43] |
| *Tockus monteiri* | Monteiros hornbill | 0 | 0.0143 | 32 | 135 | 16.62 | [44] |
| *Vireo olivaceus* | Red-eyed vireo | 57.9 | 0.0000 |  |  |  | [45] |
| *Zonotrichia albicollis* | White-throated sparrow | 31.8 | 0.0130 |  |  |  | [46] |
| *Zosterops lateralis* | Capricorn silvereye | 0 | 0.0000 |  |  |  | [47] |

**Supplementary Table 1**: source data references. Numbers equate to numbers in the Table under ‘Refs’.

1. Blomqvist D, Fessl B, Hoi H, Kleindorfer S: **High frequency of extra-pair fertilisations in the moustached warbler, a songbird with a variable breeding system.** *Behaviour* 2005, **142:**1133-1148.

2. Mee A, Whitfield DP, Thompson DBA, Burke T: **Extrapair paternity in the common sandpiper, *Actitis hypoleucos*, revealed by DNA fingerprinting.** *Anim Behav* 2004, **67:**333-342.

3. Birks SM: **Paternity in the Australian brush-turkey, *Alectura lathami*, a megapode bird with uniparental care.** *Behav Ecol* 1997, **8:**560-568.

4. Reyer H-U, Bollman K, Schläpfer AR, Schymainda A, Klecack G: **Ecological determinants of extrapair fertilizations and egg dumping in alpine water pipits (*Anthus spinoletta*).** *Behav Ecol* 1996, **8:**534-543.

5. Quinn JS, Woolfenden GE, Fitzpatrick JW, White BN: **Multi-locus DNA fingerprinting supports genetic monogamy in Florida scrub-jays.** *Behav Ecol Sociobiol* 1999, **45:**1-10.

6. Muller W, Epplen JT, Lubjuhn T: **Genetic paternity analyses in little owls (*Athene noctua*): does the high rate of paternal care select against extra-pair young?** *J Ornithol* 2001, **142:**195-203.

7. Blomqvist D, Kempenaers B, Lanctot RB, Sandercock BK: **Genetic parentage and mate guarding in the Arctic-breeding western sandpiper.** *Auk* 2002, **119:**228-233.

8. Rabouam C, Bretagnolle V, Bigot Y, Periquet G: **Genetic relationships of Cory's shearwater: parentage, mating assortment, and geographic differentiation revealed by DNA fingerprinting.** *Auk* 2000, **117:**651-662.

9. Gissing GJ, Crease TJ, Middleton ALA: **Extrapair paternity associated with renesting in the American goldfinch.** *Auk* 1998, **115:**230-234.

10. Hill GE, Montgomerie R, Roeder C, Boag PT: **Sexual selection and cockoldry in a monogamous songbird: implications for sexual selection theory.** *Behav Ecol Sociobiol* 1994, **35:**193-199.

11. Küpper C, Kis J, Kosztolányi A, Székely T, Cuthill IC, Blomqvist D: **Genetic mating system and timing of extra-pair fertilizations in the Kentish plover.** *Behav Ecol Sociobiol* 2004, **57:**32-39.

12. Wallander J, Blomqvist D, Lifjeld JT: **Genetic and social monogamy - does it occur without mate guarding in the ringed plover?** *Ethology* 2001, **107:**561-572.

13. Zharikov Y, Nol E: **Copulation behaviour, mate guarding, and paternity in the semipalmated plover.** *Condor* 2000, **102:**231-235.

14. Henderson IG, Hart PJB, Burke T: **Strict monogamy in a semi-colonial passerine: the jackdaw *Corvus monedula*.** *J Avian Biol* 2000, **31:**177-182.

15. Masello JF, Sramkova A, Quillfeldt P, Epplen JT, Lubjuhn T: **Genetic monogamy in burrowing parrots *Cyanoliseus patagonus*?** *J Avian Biol* 2002, **33:**99-103.

16. Friedl TWP, Klump GM: **Determinants of male mating success in the red bishop (*Euplectes orix*).** *Behav Ecol Sociobiol* 1999, **46:**387-399.

17. Negro JJ, Villarroel M, Tella JL, Kunhlein U, Hiraldo F, Donazar JA, Bird DM: **DNA fingerprinting reveals a low incidence of extra-pair fertilizations in the lesser kestrel.** *Anim Behav* 1996, **51:**935-943.

18. Villarroel M, Bird DM, Kuhnlein U: **Copulatory behaviour and paternity in the American kestrel: the adaptive significance of frequent copulations.** *Anim Behav* 1998, **56:**289-299.

19. Dearborn DC, Anders AD, Parker PG: **Sexual dimorphism, extrapair fertilisations, and operational sex ratio in great frigatebirds (*Fregata minor*).** *Behav Ecol* 2001, **12:**746-752.

20. Hall ML, Masgrath RD: **Duetting and mate-guarding in Australian magpie-larks (*Grallina cyanoleuca*).** *Behav Ecol Sociobiol* 2000, **47:**180-187.

21. Valera F, Hoi H, Kristín A: **Male shrikes punish unfaithful females.** *Behav Ecol* 2003, **14:**403-408.

22. Conrad KF, Clarke MF, Robertson RJ, Boag PT: **Paternity and the relatedness of helpers in the cooperatively breeding bell miner.** *Condor* 1998, **100:**343-349.

23. Hartley IR, Shepherd M, Robson T, Burke T: **Reproductive success of ploygynous male corn buntings (*Miliaria calandra*) as confirmed by DNA fingerprinting.** *Behav Ecol* 1993, **4:**310-317.

24. Quillfeldt P, Schmoll T, Peter H-U, Epplen JT, Lubjuhn T: **Genetic monogamy in Wilson's storm petrel.** *Auk* 2001, **118:**242-248.

25. Mauck RA, Waite TA, Parker PG: **Monogamy in Leach's storm petrel: DNA fingerprinting evidence.** *Auk* 1995, **112:**473-482.

26. Lawless SG, Ritchison G, Klatt PH, Westneat DF: **The mating strategies of eastern screech-owls: a genetic analysis.** *Condor* 1997, **99:**213-217.

27. Arsenault DP, Stacey PB, Hoelzer GA: **No extra-pair fertilization in flammulated owls despite aggregated nesting.** *Condor* 2002, **104:**197-201.

28. Hoi H, Hoi-Leitner M: **An alternative route to coloniality in the bearded tit: females pursue extra-pair fertilizations.** *Behav Ecol* 1997, **8:**115-119.

29. Lubjuhn T, Gerken T, Brün J, Epplen JT: **High frequency of extra-pair paternity in the coal tit.** *J Avian Biol* 1999, **30:**229-233.

30. Otter K, Ratcliffe L, Boag PT: **Extra-pair paternity in the black-capped chickadee.** *Condor* 1994, **96:**218-222.

31. Orell M, Rytkönen S, Launonen V, Welling P, Koivula K, Kimpulainen K, Bachmann L: **Low frequency of extra-pair paternity in the Willow Tit *Parus montanus* as revealed by DNA fingerprinting.** *Ibis* 1997, **139:**562-566.

32. Chu M, Koenig WD, Godinez A, McIntosh CE, Fleischer RC: **Social and genetic monogamy in territorial and loosely colonial populations of phainopepla (*Phainopepla nitens*).** *Auk* 2002, **119:**770-777.

33. Huyvaert KP, Anderson DJ, HJones TC, Duan W, Parker PG: **Extra-pair paternity in waved albatrosses.** *Mol Ecol* 2000, **9:**1415-1419.

34. Schamel D, Tracy DM, Lank DB, Westneat DF: **Mate guarding, copulation strategies and paternity in the sex-role reversed, sexually polyandrous red-necked phalarope *Phalaropus lobatus*.** *Behav Ecol Sociobiol* 2004, **57:**110-118.

35. Moreno J, Boto L, Fargallo JA, de Léon A, Potti J: **Absence of extra-pair fertilisations in the chinstrap penguin *Pygoscelis antarctica*.** *J Avian Biol* 2000, **31:**580-583.

36. Schleicher B, Hoi H, Valera F, Hoi-Leitner M: **The importance of different paternity guards in the polygynandrous penduline tit (*Remiz pendulinus*).** *Behav* 1997, **134:**941-959.

37. Vogt C, Leitner S, Gahr M: **Mate fidelity in a population of island canaries (*Serinus canaria*) in the Madeiran archipelago.** *J Ornithol* 2003, **144:**86-92.

38. Hoi-Leitner M, Hoi H, Romero-Pujante M, Valera F: **Female extra-pair behaviour and environmental quality in the serin (*Serinus serinus*): a test of the 'constrained female hypothesis'.** *Proc Roy Soc Lond B* 1999, **266:**1021-1026.

39. Perreault S, Lemon RE, Kuhnlein U: **Patterns and corrlates of extrapair paternity in American redstarts (*Setophaga ruticilla*).** *Behav Ecol* 1997, **8:**612-621.

40. Griggio M, Matessi G, Marin G: **No evidence of extra-pair paternity in a colonial seabird, the common tern (*Sterna hirundo*).** *Ital J Zool* 2004, **71:**219-222.

41. Cordero PJ, Veiga JP, Moreno J, Parkin DT: **Extra-pair paternity in the facultatively polygynous spotless starling, *Sturnus unicolor*.** *Behav Ecol Sociobiol* 2003, **54:**1-6.

42. Lorensten S-H, Amundsen T, Athonisen K, Lifjeld JT: **Molecular evidence for extrapair paternity and female-female pairs in antarctic petrels.** *Auk* 2000, **117:**1042-1047.

43. Haggerty TM, Morton ES, Fleischer RC: **Genetic monogamy in Carolina wrens (*Thyothorus ludovicianus*).** *Auk* 2001, **118:**215-219.

44. Stanback M, Richardson DS, Boix-Hinzen C, Mendelsohn J: **Genetic monogamy in Monteiro's hornbill, *Tockus monteiri*.** *Anim Behav* 2002, **63:**787-793.

45. Morton ES, Stutchbury BJM, Howlett JS, Piper WH: **Genetic monogamy in blue-headed vireos and a comparison with a sympatric vireo with extrapair paternity.** *Behav Ecol* 1998, **9:**515-524.

46. Tuttle EM: **Alternative reproductive strategies in the white-throated sparrow: behavioural and genetic evidence.** *Behav Ecol* 2003, **14:**425-432.

47. Robertson BC, Degnan SM, Kikkawa J, Moritz CC: **Genetic monogamy in the absence of paternity guards: the Capricorn silvereye, Zosterops lateralis chlorocephalus, on Heron Island.** *Behav Ecol* 2001, **12:**666-673.
