# Supplementary material for: Sexual selection does not influence minisatellite mutation rate
Source: BMC Evol Biol. 2009 Jan 8;9:5. doi: 10.1186/1471-2148-9-5 (PMC2636768; doi:10.1186/1471-2148-9-5)
Supplement: Additional file 2 — Response Supplementary table 1. Information on species, research laboratory, number of individuals with 0, 1, 2, 3, 4, 5 or 6 novel bands due to mutations, mean number of bands scored (always from nestlings if there were data for both adults and nestlings), number of offspring used for mutation estimation, total number of offspring, estimated mutation rate, mutation rate as reported in the publication, extra-pair paternity (%; if there was information based on more than one probe, then the estimate based on the largest sample size), genetic marker (only that/those used for estimating mutation rates), whether the molecular marker was a minisatellite, whether the study was included by Amos [1], and reference. References are listed in Møller & Cuervo [2] and Amos [1], and if not included there, the references are listed below. [file 1471-2148-9-5-S2.doc]

Supplementary Table 1. Information on species, research laboratory, number of individuals with 0, 1, 2, 3, 4, 5 or 6 novel bands due to mutations, mean number of bands scored (always from nestlings if there were data for both adults and nestlings), number of offspring used for mutation estimation, total number of offspring, estimated mutation rate, mutation rate as reported in the publication, extra-pair paternity (%; if there was information based on more than one probe, then the estimate based on the largest sample size), genetic marker (only that/those used for estimating mutation rates), whether the molecular marker was a minisatellite, whether the study was included by Amos [1], and reference. References are listed in Møller & Cuervo [2] and Amos [1], and if not included there, the references are listed below.

| Species | Research laboratory | No. individuals with 0 novel bands | No. individuals with 1 novel band | No. individuals with 2 novel bands | No. individuals with 3 novel bands | No. individuals with 4 novel bands | No. individuals with 5 novel bands | No. individuals with 6 novel bands | Mean no. bands scored |
| --- | --- | --- | --- | --- | --- | --- | --- | --- | --- |
| *Acrocephalus arundinaceus* | 1 | 484 | 48 | 4 | 0 | 0 | 0 | 0 | 27.3 |
| *Acrocephalus melanopogon* | 22 | 13 | 5 | 0 | 0 | 0 | 0 | 0 | 17.9 |
| *Acrocephalus schoenobaenus* | 1 | 115 | 27 | 1 | 0 | 0 | 0 | 0 | 33 |
| *Acrocephalus schoenobaenus* | 34 | 81 | 28 | 7 | 1 | 0 | 0 | 0 | 61.1 |
| *Acrocephalus vaughani* | 2 | 20 | 0 | 0 | 0 | 0 | 0 | 0 | . |
| *Actitis hypoleucos* | 2 | 45 | 10 | 5 | 0 | 0 | 0 | 0 | 47.1 |
| *Agelaius phoeniceus* | 3 | 104 | 47 | 18 | 6 | 1 | 0 | 0 | 71.6 |
| *Agelaius phoeniceus* | 4 | . | . | . | . | . | . | . | 30.5 |
| *Alectura lathami* | 51 | 35 | 5 | 0 | 0 | 0 | 0 | 0 | 26.2 |
| *Anser caerulescens* | 9 | 74 | 0 | 1 | 0 | 0 | 0 | 0 | 15.7 |
| *Anser rossi* | 9 | 76 | 5 | 0 | 0 | 0 | 0 | 0 | 20 |
| *Anthus spinoletta* | 52 | 96 | 125 | 191 | 164 | 86 | 19 | 0 | 27.2 |
| *Aphelocoma coerulescens* | 35 | 110 | 27 | 2 | 0 | 0 | 0 | 0 | 40.5 |
| *Apus apus* | 5 | 62 | 14 | 4 | 0 | 0 | 0 | 0 | 19.4 |
| *Asio otus* | 8 | 56 | 3 | 0 | 0 | 0 | 0 | 0 | 21.5 |
| *Athene noctua* | 16 | 49 | 4 | 0 | 0 | 0 | 0 | 0 | 20.95 |
| *Branta leucopsis* | 51 | 8 | 5 | 1 | 0 | 0 | 0 | 0 | 25.8 |
| *Branta leucopsis* | 51 | 8 | 5 | 1 | 0 | 0 | 0 | 0 | 22.1 |
| *Branta leucopsis* | 6 | 112 | 2 | 0 | 0 | 0 | 0 | 0 | 21.4 |
| *Buteo galapagoensis* | 36 | 18 | 1 | 0 | 0 | 0 | 0 | 0 | 37.9 |
| *Calidris maritima* | 7 | 66 | 14 | 1 | 0 | 0 | 0 | 0 | 25.6 |
| *Calidris mauri* | 22 | 41 | 11 | 5 | 0 | 0 | 0 | 0 | 32 |
| *Calonectris diomedea* | 37 | 29 | 3 | 2 | 0 | 0 | 0 | 0 | 26.7 |
| *Campylorhynchus griseus* | 8 | 176 | 30 | 1 | 0 | 0 | 0 | 0 | 33 |
| *Campylorhynchus griseus* | 8 | . | . | . | . | . | . | . | . |
| *Campylorhynchus nuchalis* | 8 | 58 | 10 | 0 | 0 | 0 | 0 | 0 | 54.4 |
| *Cardinalis cardinalis* | 3 | 21 | 9 | 2 | 0 | 0 | 0 | 0 | 29.6 |
| *Cardinalis cardinalis* | 3 | 21 | 9 | 2 | 0 | 0 | 0 | 0 | 38.1 |
| *Carduelis tristis* | 53 | 42 | 10 | 2 | 0 | 0 | 0 | 0 | 20 |
| *Carpodacus mexicanus* | 9 | 71 | 2 | 0 | 0 | 0 | 0 | 0 | 22.5 |
| *Catharacta lonnbergi* | 52 | . | . | . | . | . | . | . | 29.3 |
| *Catharacta maccormicki* | 52 | 11 | 1 | 0 | 0 | 0 | 0 | 0 | 26.85 |
| *Cercomacra tyrannina* | 11 | 4 | 5 | 2 | 0 | 0 | 0 | 0 | 35 |
| *Charadrius alexandrinus* | 22 | 118 | 43 | 6 | 0 | 0 | 0 | 0 | 18 |
| *Charadrius hiaticula* | 22 | 38 | 9 | 3 | 0 | 0 | 0 | 0 | 17.9 |
| *Charadrius semipalmatus* | 12 | 60 | 2 | 0 | 0 | 0 | 0 | 0 | 27.3 |
| *Coragyps atratus* | 8 | 33 | 3 | 0 | 0 | 0 | 0 | 0 | 21.7 |
| *Coragyps atratus* | 8 | 32 | 3 | 1 | 0 | 0 | 0 | 0 | 22.8 |
| *Corcorax melanoramphos* | 19 | 37 | 12 | 2 | 0 | 0 | 0 | 0 | 39.9 |
| *Corvus monedula* | 2 | . | . | . | . | . | . | . | 15.41 |
| *Cyanoliseus patagonus* | 23 | 151 | 12 | 1 | 0 | 0 | 0 | 0 | 25.6 |
| *Cyanopica cyanus* | 13 | 23 | 7 | 0 | 0 | 0 | 0 | 0 | 20.47 |
| *Dacelo novaeguineae* | 19 | 130 | 8 | 2 | 0 | 0 | 0 | 0 | 37.2 |
| *Delichon urbica* | 14 | 54 | 7 | 0 | 0 | 0 | 0 | 0 | 14.5 |
| *Delichon urbica* | 7 | 57 | 2 | 0 | 0 | 0 | 0 | 0 | 23.6 |
| *Dendroica caerulescens* | 31 | 74 | 15 | 3 | 0 | 0 | 0 | 0 | 15 |
| *Dendroica petechia* | 9 | 101 | 19 | 3 | 0 | 0 | 0 | 0 | 34.73 |
| *Euplectes orix* | 38 | 116 | 29 | 0 | 0 | 0 | 0 | 0 | 10.6 |
| *Falco columbarius* | 10 | 18 | 12 | 1 | 0 | 0 | 0 | 0 | 19.46 |
| *Falco columbarius* | 10 | 24 | 7 | 0 | 0 | 0 | 0 | 0 | 17.12 |
| *Falco naumanni* | 15 | 64 | 3 | 0 | 0 | 0 | 0 | 0 | 10.9 |
| *Falco sparverius* | 16 | 59 | 15 | 5 | 0 | 0 | 0 | 0 | 12.2 |
| *Ficedula hypoleuca* | 16 | 146 | 10 | 0 | 0 | 0 | 0 | 0 | 21.7 |
| *Ficedula hypoleuca* | 6 | 191 | 5 | 2 | 1 | 0 | 0 | 0 | 19 |
| *Ficedula hypoleuca* | 6 | 8 | 2 | 0 | 0 | 0 | 0 | 0 | 13.8 |
| *Ficedula hypoleuca* | 6 | 23 | 3 | 0 | 0 | 0 | 0 | 0 | 15.2 |
| *Ficedula hypoleuca* | 7 | 125 | 4 | 0 | 0 | 0 | 0 | 0 | 11.5 |
| *Fregata minor* | 8 | 71 | 19 | 1 | 0 | 0 | 0 | 0 | 14.1 |
| *Fringilla coelebs* | 2 | 17 | 3 | 0 | 0 | 0 | 0 | 0 | 23.81 |
| *Fulmarus glacialis* | 2 | . | . | . | . | . | . | . | . |
| *Gallinula chloropus* | 2 | . | . | . | . | . | . | . | . |
| *Gallinula chloropus* | 2 | . | . | . | . | . | . | . | . |
| *Gavia immer* | 11 | 53 | 2 | 0 | 0 | 0 | 0 | 0 | 17 |
| *Geothlypis trichas* | 9 | 71 | 1 | 0 | 0 | 0 | 0 | 0 | 16 |
| *Grallina cyanoleuca* | 19 | 95 | 5 | 0 | 0 | 0 | 0 | 0 | 11.3 |
| *Grallina cyanoleuca* | 19 | 41 | 3 | 0 | 0 | 0 | 0 | 0 | 11.3 |
| *Haematopus ostralegus* | 2 | 60 | 4 | 0 | 0 | 0 | 0 | 0 | 22 |
| *Hirundo rustica* | 6 | 178 | 10 | 0 | 0 | 0 | 0 | 0 | 20.3 |
| *Hymenolaimus malacorhynchos* | 17 | 13 | 1 | 0 | 0 | 0 | 0 | 0 | 43.6 |
| *Lagopus lagopus* | 53 | . | . | . | . | . | . | . | . |
| *Lanius bucephalus* | 18 | 81 | 6 | 2 | 0 | 0 | 0 | 0 | 46.2 |
| *Lanius minor* | 22 | 120 | 14 | 2 | 0 | 0 | 0 | 0 | 21 |
| *Larus occidentalis* | 2 | 6 | 6 | 1 | 0 | 0 | 0 | 0 | 25.2 |
| *Larus occidentalis* | 2 | 8 | 8 | 0 | 0 | 0 | 0 | 0 | 21.7 |
| *Loxoides bailleui* | 11 | 19 | 1 | 0 | 0 | 0 | 0 | 0 | 17.9 |
| *Loxoides bailleui* | 11 | 13 | 7 | 0 | 0 | 0 | 0 | 0 | 12.2 |
| *Luscinia svecica* | 7 | . | . | . | . | . | . | . | 29.9 |
| *Malurus cyaneus* | 19 | 33 | 9 | 1 | 0 | 0 | 0 | 0 | 36 |
| *Manorina melanophrys* | 9 | 2 | 4 | 7 | 6 | 3 | 0 | 1 | 15.28 |
| *Melanerpes formicivorus* | 20 | 41 | 8 | 1 | 0 | 0 | 0 | 0 | 15 |
| *Merops apiaster* | 21 | 46 | 5 | 0 | 0 | 0 | 0 | 0 | 23.7 |
| *Miliaria calandra* | 2 | 32 | 4 | 0 | 0 | 0 | 0 | 0 | 13.4 |
| *Notiomystis cincta* | 29 | 18 | 4 | 0 | 0 | 0 | 0 | 0 | 19.53 |
| *Notiomystis cincta* | 29 | 20 | 2 | 0 | 0 | 0 | 0 | 0 | 18.63 |
| *Oceanites oceanicus* | 39 | 51 | 12 | 0 | 0 | 0 | 0 | 0 | 38.1 |
| *Oceanodroma leucorhoa* | 8 | 35 | 7 | 0 | 0 | 0 | 0 | 0 | 21.4 |
| *Oenanthe oenanthe* | 2 | 48 | 6 | 0 | 0 | 0 | 0 | 0 | 27.8 |
| *Otus asio* | 3 | 53 | 18 | 5 | 0 | 0 | 0 | 0 | 51.4 |
| *Otus flammeolus* | 54 | 32 | 5 | 0 | 0 | 0 | 0 | 0 | 44 |
| *Panurus biarmicus* | 22 | 129 | 18 | 1 | 0 | 0 | 0 | 0 | 26 |
| *Parus ater* | 16 | 103 | 14 | 1 | 0 | 0 | 0 | 0 | 14.03 |
| *Parus atricapillus* | 9 | 8 | 30 | 6 | 0 | 0 | 0 | 0 | 20.26 |
| *Parus caeruleus* | 23 | . | . | . | . | . | . | . | 20 |
| *Parus caeruleus* | 23 | 740 | 31 | 0 | 0 | 0 | 0 | 0 | 18.2 |
| *Parus caeruleus* | 6 | 44 | 4 | 0 | 0 | 0 | 0 | 0 | 29.4 |
| *Parus cristatus* | 23 | . | . | . | . | . | . | . | 19 |
| *Parus major* | 24 | 455 | 20 | 0 | 0 | 0 | 0 | 0 | 27.9 |
| *Parus major* | 24 | 471 | 9 | 0 | 0 | 0 | 0 | 0 | 33.4 |
| *Parus major* | 6 | 34 | 4 | 0 | 2 | 0 | 0 | 0 | 31.4 |
| *Parus montanus* | 25 | 92 | 12 | 6 | 1 | 0 | 0 | 0 | 19.5 |
| *Passer domesticus* | 10 | 18 | 0 | 0 | 0 | 0 | 0 | 0 | 14.25 |
| *Passer domesticus* | 2 | 9 | 1 | 0 | 0 | 0 | 0 | 0 | 28.8 |
| *Passer domesticus* | 40 | 131 | 9 | 0 | 0 | 0 | 0 | 0 | 12.98 |
| *Passer domesticus* | 41 | 81 | 23 | 4 | 0 | 1 | 0 | 0 | 38.6 |
| *Passer domesticus* | 10 | 366 | 3 | 0 | 0 | 0 | 0 | 0 | 12.88 |
| *Passer domesticus* | 22 | 80 | 9 | 7 | 0 | 0 | 0 | 0 | 29.7 |
| *Passerculus sandwichensis* | 26 | . | . | . | . | . | . | . | 16 |
| *Passerina cyanea* | 3 | 28 | 10 | 3 | 0 | 0 | 0 | 0 | 37.5 |
| *Perisoreus infaustus* | 6 | 20 | 0 | 0 | 0 | 0 | 0 | 0 | 19 |
| *Petroica australis* | 29 | . | . | . | . | . | . | . | 34.9 |
| *Petroica australis* | 29 | 27 | 4 | 0 | 0 | 0 | 0 | 0 | 37 |
| *Petronia petronia* | 42 | . | . | . | . | . | . | . | 23.9 |
| *Phainopepla nitens* | 31 | 28 | 6 | 0 | 0 | 0 | 0 | 0 | 27.8 |
| *Phalacrocorax aristotelis* | 27 | 21 | 2 | 0 | 0 | 0 | 0 | 0 | 16.07 |
| *Phalaropus lobatus* | 3 | 93 | 22 | 14 | 6 | 5 | 0 | 0 | 51.1 |
| *Phalaropus tricolor* | 9 | 33 | 9 | 1 | 0 | 0 | 0 | 0 | 14.2 |
| *Phoebastria irrorata* | 8 | 11 | 1 | 0 | 0 | 0 | 0 | 0 | 15.8 |
| *Phoebastria irrorata* | 8 | 123 | 5 | 0 | 0 | 0 | 0 | 0 | 15.1 |
| *Phylloscopus trochilus* | 7 | 53 | 15 | 5 | 0 | 0 | 0 | 0 | 28.6 |
| *Pica pica* | 2 | 10 | 6 | 1 | 0 | 0 | 0 | 0 | 21 |
| *Picoides borealis* | 28 | 79 | 0 | 0 | 0 | 0 | 0 | 0 | 45.1 |
| *Porphyrio hochstetteri* | 43 | 27 | 0 | 0 | 0 | 0 | 0 | 0 | 12.15 |
| *Porphyrio porphyrio* | 29 | . | . | . | . | . | . | . | . |
| *Progne subis* | 30 | . | . | . | . | . | . | . | 17 |
| *Progne subis* | 30 | 31 | 3 | 0 | 0 | 0 | 0 | 0 | 21.9 |
| *Prunella collaris* | 2 | 55 | 10 | 0 | 0 | 0 | 0 | 0 | 27.9 |
| *Prunella modularis* | 2 | 129 | 2 | 1 | 0 | 0 | 0 | 0 | 23.2 |
| *Psaltriparus minimus* | 35 | . | . | . | . | . | . | . | 19.1 |
| *Puffinus tenuirostris* | 10 | 7 | 0 | 0 | 0 | 0 | 0 | 0 | 15.7 |
| *Pygoscelis adeliae* | 42 | 15 | 3 | 2 | 0 | 0 | 0 | 0 | 18 |
| *Pygoscelis antarctica* | 55 | 70 | 6 | 0 | 0 | 0 | 0 | 0 | 10.6 |
| *Remiz pendulinus* | 22 | 64 | 34 | 17 | 0 | 0 | 0 | 0 | 11.4 |
| *Riparia riparia* | 14 | 124 | 9 | 2 | 1 | 0 | 0 | 0 | 20 |
| *Riparia riparia* | 22 | 78 | 35 | 16 | 2 | 0 | 0 | 0 | 21.2 |
| *Sayornis phoebe* | 9 | 57 | 9 | 1 | 0 | 0 | 0 | 0 | 41.25 |
| *Sericornis frontinalis* | 9 | 112 | 8 | 0 | 0 | 0 | 0 | 0 | 30 |
| *Serinus canaria* | 23 | 43 | 2 | 0 | 0 | 0 | 0 | 0 | 24.1 |
| *Serinus serinus* | 22 | . | . | . | . | . | . | . | . |
| *Serinus serinus* | 22 | 109 | 14 | 1 | 0 | 0 | 0 | 0 | 21 |
| *Setophaga ruticilla* | 31 | 57 | 5 | 0 | 0 | 0 | 0 | 0 | 15.4 |
| *Sialia mexicanus* | 20 | . | . | . | . | . | . | . | . |
| *Sialia sialis* | 9 | 17 | 4 | 0 | 0 | 0 | 0 | 0 | 20.98 |
| *Spheniscus humboldti* | 11 | 40 | 8 | 1 | 0 | 0 | 0 | 0 | 36.4 |
| *Sterna hirundo* | 56 | 19 | 3 | 2 | 0 | 0 | 0 | 0 | 20.5 |
| *Sturnus unicolor* | 44 | 248 | 20 | 2 | 0 | 0 | 0 | 0 | 17.06 |
| *Sturnus vulgaris* | 2 | 47 | 3 | 1 | 0 | 0 | 0 | 0 | 31.6 |
| *Sturnus vulgaris* | 2 | 45 | 5 | 1 | 0 | 0 | 0 | 0 | 30.9 |
| *Sturnus vulgaris* | 32 | 81 | 2 | 1 | 0 | 0 | 0 | 0 | 16 |
| *Tachycineta bicolor* | 9 | 43 | 9 | 1 | 0 | 0 | 0 | 0 | 30.9 |
| *Tachycineta bicolor* | 9 | 32 | 3 | 1 | 0 | 0 | 0 | 0 | 30.9 |
| *Tachycineta bicolor* | 9 | 4 | 4 | 7 | 6 | 7 | 0 | 0 | 50 |
| *Taeniopygia guttata* | 2 | 75 | 4 | 1 | 0 | 0 | 0 | 0 | 22.8 |
| *Tetrao tetrix* | 2 | 45 | 17 | 0 | 0 | 0 | 0 | 0 | 33.6 |
| *Thalassoica antarctica* | 7 | 27 | 11 | 0 | 0 | 0 | 0 | 0 | 30.9 |
| *Thryothorus ludovicianus* | 45 | 68 | 13 | 2 | 1 | 0 | 0 | 0 | 13.3 |
| *Tockus monteiri* | 46 | 109 | 20 | 6 | 0 | 0 | 0 | 0 | 16.62 |
| *Tribonyx mortierii* | 9 | . | . | . | . | . | . | . | . |
| *Troglodytes aedon* | 33 | 710 | 27 | 4 | 1 | 0 | 0 | 0 | 37.5 |
| *Turdoides squamiceps* | 8 | 178 | 8 | 0 | 0 | 0 | 0 | 0 | 64.1 |
| *Turdus grayi* | 30 | 18 | 3 | 0 | 0 | 0 | 0 | 0 | 15.7 |
| *Tyrannus tyrannus* | 47 | 24 | 12 | 1 | 0 | 0 | 0 | 0 | 14 |
| *Tyto alba* | 16 | 105 | 16 | 0 | 0 | 0 | 0 | 0 | 26.8 |
| *Tyto alba* | 16 | 89 | 0 | 0 | 0 | 0 | 0 | 0 | 8.35 |
| *Upupa epops* | 48 | 68 | 21 | 8 | 2 | 0 | 0 | 0 | 19.4 |
| *Uria aalge* | 2 | . | . | . | . | . | . | . | . |
| *Vireo solitarius* | 30 | 36 | 0 | 0 | 0 | 0 | 0 | 0 | 16.4 |
| *Wilsonia citrina* | 11 | . | . | . | . | . | . | . | 14.3 |
| *Wilsonia citrina* | 30 | 41 | 8 | 6 | 0 | 0 | 0 | 0 | 23 |
| *Zonotrichia albicollis* | 49 | . | . | . | . | . | 0 | 0 | . |
| *Zosterops lateralis* | 50 | 54 | 0 | 0 | 0 | 0 | 0 | 0 | 13.7 |

| Species | No. offspring used for mutation estimate | Total number of offspring | Estimated mutation rate | Mutation rate in publication | Extra-pair paternity (%) | Genetic marker | Genetic marker is a minisatellite | Study included in Amos [1] | Reference |
| --- | --- | --- | --- | --- | --- | --- | --- | --- | --- |
| *Acrocephalus arundinaceus* | 536 | 678 | 0.003827 | . | 3.07 | 33.15 | 1 | 0 |  |
| *Acrocephalus melanopogon* | 18 | 44 | 0.015518 | . | 27.27 | per | 1 | 0 |  |
| *Acrocephalus schoenobaenus* | 143 | 201 | 0.006145 | . | 7.46 | 33.15, 33.6 | 1 | 0 |  |
| *Acrocephalus schoenobaenus* | 117 | 143 | 0.006295 | 0.008000 | 8.39 | 33.15, 33.6 | 1 | 1 | [1] |
| *Acrocephalus vaughani* | 20 | 42 | . | 0.000000 | 0.00 | 33.15 | 1 | 0 |  |
| *Actitis hypoleucos* | 60 | 80 | 0.007077 | 0.006000 | 15.66 | 33.6, 33.15 | 1 | 0 |  |
| *Agelaius phoeniceus* | 176 | 235 | 0.008332 | 0.008000 | 24.68 | M13, M2.5, 18.15 | 1 | 0 |  |
| *Agelaius phoeniceus* | 267 | 403 | . | 0.013910 | 33.75 | M13, per, 33.6 | 1 | 0 |  |
| *Alectura lathami* | 50 | 65 | 0.003817 | 0.003333 | 27.69 | 18.15 | 1 | 0 |  |
| *Anser caerulescens* | 75 | 80 | 0.001699 | . | 5.00 | per, M13 | 1 | 0 |  |
| *Anser rossi* | 81 | 83 | 0.003086 | . | 2.41 | per, M13 | 1 | 0 |  |
| *Anthus spinoletta* | 681 | 1052 | 0.077632 | . | 5.23 | 33.15 | 1 | 0 |  |
| *Aphelocoma coerulescens* | 139 | 139 | 0.005507 | 0.005000 | 0.00 | 33.15, pSP2.5R1 | 1 | 0 |  |
| *Apus apus* | 84 | 88 | 0.013500 | 0.013000 | 4.55 | 33.15 | 1 | 0 |  |
| *Asio otus* | 59 | 59 | 0.002365 | 0.001800 | 0.00 | 33.15 | 1 | 0 |  |
| *Athene noctua* | 53 | 53 | 0.003602 | 0.003500 | 0.00 | (CA)8 | 0 | 0 |  |
| *Branta leucopsis* | 14 | 18 | 0.019380 | 0.013800 | 0.00 | 33.15 | 1 | 0 |  |
| *Branta leucopsis* | 14 | 18 | 0.022624 | 0.006500 | 0.00 | 33.6 | 1 | 0 |  |
| *Branta leucopsis* | 114 | 137 | 0.000820 | 0.000800 | 0.00 | 33.15 | 1 | 0 |  |
| *Buteo galapagoensis* | 19 | 19 | 0.001389 | 0.001200 | 0.00 | 33.15, 33.6 | 1 | 1 | [2] |
| *Calidris maritima* | 81 | 82 | 0.007716 | 0.008000 | 1.22 | per | 1 | 0 |  |
| *Calidris mauri* | 57 | 98 | 0.011513 | . | 5.10 | per | 1 | 0 |  |
| *Calonectris diomedea* | 34 | 34 | 0.007711 | 0.007000 | 0.00 | (GGAT)4 | 0 | 0 |  |
| *Campylorhynchus griseus* | 207 | 222 | 0.004685 | 0.004700 | 2.30 | 33.6, 33.15 | 1 | 0 |  |
| *Campylorhynchus griseus* | 207 | 222 | . | 0.003900 | 2.30 | 33.6, 33.15 | 1 | 0 |  |
| *Campylorhynchus nuchalis* | 68 | 69 | 0.002703 | . | 1.40 | 33.6, 33.15 | 1 | 0 |  |
| *Cardinalis cardinalis* | 32 | 37 | 0.013725 | . | 13.51 | M13 | 1 | 0 |  |
| *Cardinalis cardinalis* | 32 | 37 | 0.010663 | . | 13.51 | per | 1 | 0 |  |
| *Carduelis tristis* | 54 | 70 | 0.012963 | . | 14.29 | 33.15 | 1 | 0 |  |
| *Carpodacus mexicanus* | 73 | 119 | 0.001218 | . | 8.40 | 33.15, per | 1 | 0 |  |
| *Catharacta lonnbergi* | 45 | 45 | . | 0.001400 | 0.00 | 33.15, 33.6 | 1 | 0 |  |
| *Catharacta maccormicki* | 12 | 14 | 0.003104 | . | 7.14 | 33.15 | 1 | 0 |  |
| *Cercomacra tyrannina* | 11 | 15 | 0.023377 | . | 0.00 | 33.15, 33.6 | 1 | 0 |  |
| *Charadrius alexandrinus* | 167 | 229 | 0.018297 | 0.015000 | 1.31 | per | 1 | 0 |  |
| *Charadrius hiaticula* | 50 | 57 | 0.016760 | . | 0.00 | 33.15 | 1 | 0 |  |
| *Charadrius semipalmatus* | 62 | 85 | 0.001182 | 0.001200 | 4.71 | 33.15 | 1 | 0 |  |
| *Coragyps atratus* | 36 | 36 | 0.003840 | 0.004000 | 0.00 | 33.6, 33.15, M13 | 1 | 0 |  |
| *Coragyps atratus* | 36 | 36 | 0.006092 | 0.004000 | 0.00 | 33.6, 33.15, M13 | 1 | 0 |  |
| *Corcorax melanoramphos* | 51 | 51 | 0.007863 | . | 0.00 | per, 33.15 | 1 | 1 | [3] |
| *Corvus monedula* | 74 | 74 | . | 0.004386 | 0.00 | 33.15, 33.6 | 1 | 0 |  |
| *Cyanoliseus patagonus* | 164 | 166 | 0.003335 | 0.003000 | 0.00 | (CA)8 | 0 | 0 |  |
| *Cyanopica cyanus* | 30 | 30 | 0.011399 | . | 0.00 | Jeffreys 33.6 | 1 | 0 |  |
| *Dacelo novaeguineae* | 140 | 140 | 0.002304 | 0.002200 | 0.00 | 33.15, 33.6 | 1 | 1 | [4] |
| *Delichon urbica* | 61 | 72 | 0.007914 | . | 14.52 | 33.15 | 1 | 0 |  |
| *Delichon urbica* | 59 | 73 | 0.001436 | 0.006000 | 19.18 | 33.15 | 1 | 0 |  |
| *Dendroica caerulescens* | 92 | 125 | 0.015217 | 0.012000 | 27.20 | (GGAT)4 | 0 | 0 |  |
| *Dendroica petechia* | 123 | 355 | 0.005852 | 0.004600 | 36.62 | 33.15, per | 1 | 0 |  |
| *Euplectes orix* | 145 | 432 | 0.018868 | 0.019000 | 17.59 | (GGAT)4 | 0 | 0 |  |
| *Falco columbarius* | 31 | 54 | 0.023207 | . | 0.00 | 33.6 | 1 | 0 |  |
| *Falco columbarius* | 31 | 54 | 0.013190 | . | 0.00 | 33.15 | 1 | 0 |  |
| *Falco naumanni* | 67 | 147 | 0.004108 | 0.004100 | 3.45 | (GGAT)4 | 0 | 0 |  |
| *Falco sparverius* | 79 | 89 | 0.025939 | . | 11.24 | (GGAT)4 | 0 | 0 |  |
| *Ficedula hypoleuca* | 156 | 165 | 0.002954 | 0.003000 | 5.45 | CA8 | 0 | 0 |  |
| *Ficedula hypoleuca* | 199 | 223 | 0.003174 | . | 10.76 | 33.15 | 1 | 0 |  |
| *Ficedula hypoleuca* | 10 | 38 | 0.014493 | . | 23.68 | M13 | 1 | 0 |  |
| *Ficedula hypoleuca* | 26 | 38 | 0.007591 | . | 23.68 | 33.15 | 1 | 0 |  |
| *Ficedula hypoleuca* | 129 | 135 | 0.002696 | . | 4.44 | 3'HVR | 1 | 0 |  |
| *Fregata minor* | 91 | 92 | 0.016367 | 0.016400 | 1.09 | 33.15 | 1 | 0 |  |
| *Fringilla coelebs* | 20 | 47 | 0.006300 | 0.007000 | 17.02 | 33.6 | 1 | 0 |  |
| *Fulmarus glacialis* | 19 | 19 | . | 0.017000 | 0.00 | 33.15 | 1 | 0 |  |
| *Gallinula chloropus* | 90 | 90 | . | 0.002500 | 0.00 | 33.15 | 1 | 0 |  |
| *Gallinula chloropus* | 90 | 90 | . | 0.002500 | 0.00 | cGaMSO2 | 1 | 0 |  |
| *Gavia immer* | 55 | 58 | 0.002139 | . | 0.00 | 33.15 | 1 | 0 |  |
| *Geothlypis trichas* | 72 | 153 | 0.000868 | 0.001020 | 20.26 | 33.15, per | 1 | 1 | [5] |
| *Grallina cyanoleuca* | 100 | 103 | 0.004425 | . | 2.91 | per | 1 | 0 |  |
| *Grallina cyanoleuca* | 44 | 103 | 0.006034 | . | 2.91 | per | 1 | 0 |  |
| *Haematopus ostralegus* | 64 | 65 | 0.002841 | . | 1.54 | 33.6 | 1 | 0 |  |
| *Hirundo rustica* | 188 | 261 | 0.002620 | 0.002000 | 27.97 | 33.15 | 1 | 0 |  |
| *Hymenolaimus malacorhynchos* | 14 | 14 | 0.001638 | . | 0.00 | 33.15, 3'HVR | 1 | 0 |  |
| *Lagopus lagopus* | 232 | 256 | . | 0.008200 | 9.38 | per, 33.15, 33.6 | 1 | 0 |  |
| *Lanius bucephalus* | 89 | 99 | 0.002432 | 0.004400 | 10.10 | 33.6, 33.15 | 1 | 0 |  |
| *Lanius minor* | 136 | 136 | 0.006303 | . | 0.00 | (GATA)4 | 0 | 0 |  |
| *Larus occidentalis* | 13 | 33 | 0.024420 | . | 0.00 | 33.15 | 1 | 0 |  |
| *Larus occidentalis* | 16 | 33 | 0.023041 | . | 0.00 | 33.6 | 1 | 0 |  |
| *Loxoides bailleui* | 20 | 20 | 0.002793 | 0.004000 | 0.00 | M13 | 1 | 0 |  |
| *Loxoides bailleui* | 20 | 20 | 0.028689 | 0.014000 | 0.00 | 33.15 | 1 | 0 |  |
| *Luscinia svecica* | 120 | 150 | . | 0.015318 | 20.00 | 33.15 | 1 | 0 |  |
| *Malurus cyaneus* | 43 | 181 | 0.007106 | . | 76.24 | 33.15, per | 1 | 0 |  |
| *Manorina melanophrys* | 23 | 24 | 0.153654 | . | 4.17 | 33.15 | 1 | 0 |  |
| *Melanerpes formicivorus* | 50 | 51 | 0.013333 | . | 0.00 | 33.15 | 1 | 1 | [6] |
| *Merops apiaster* | 51 | 100 | 0.004137 | 0.004100 | 1.00 | 33.15 | 1 | 0 |  |
| *Miliaria calandra* | 36 | 44 | 0.008292 | 0.008264 | 4.55 | 33.6 | 1 | 0 |  |
| *Notiomystis cincta* | 22 | 34 | 0.009310 | 0.007550 | 35.29 | 33.15 | 1 | 0 |  |
| *Notiomystis cincta* | 22 | 34 | 0.004880 | 0.007550 | 35.29 | CA | 1 | 0 |  |
| *Oceanites oceanicus* | 63 | 63 | 0.004999 | 0.005000 | 0.00 | (GGAT)4 | 0 | 0 |  |
| *Oceanodroma leucorhoa* | 42 | 48 | 0.007788 | 0.008000 | 0.00 | 33.15 | 1 | 0 |  |
| *Oenanthe oenanthe* | 54 | 73 | 0.003997 | 0.003861 | 10.96 | 33.15 | 1 | 0 |  |
| *Otus asio* | 76 | 80 | 0.007168 | . | 0.00 | M13, per | 1 | 0 |  |
| *Otus flammeolus* | 37 | 37 | 0.003071 | 0.003100 | 0.00 | 33.15, 33.6 | 1 | 0 |  |
| *Panurus biarmicus* | 148 | 187 | 0.005198 | . | 14.44 | (GATA)4 | 0 | 0 |  |
| *Parus ater* | 118 | 158 | 0.009665 | 0.006300 | 25.32 | (CA)8 | 0 | 0 |  |
| *Parus atricapillus* | 44 | 53 | 0.047115 | . | 16.98 | per, 33.15 | 1 | 0 |  |
| *Parus caeruleus* | 265 | 314 | . | 0.002000 | 10.51 | 33.15 | 1 | 0 |  |
| *Parus caeruleus* | 771 | 864 | 0.002209 | . | 10.76 | 33.15 | 1 | 0 |  |
| *Parus caeruleus* | 48 | 51 | 0.002834 | 0.003000 | 5.88 | M13, 33.15 | 1 | 0 |  |
| *Parus cristatus* | 106 | 121 | . | 0.009000 | 12.40 | 33.15 | 1 | 0 |  |
| *Parus major* | 475 | 516 | 0.001509 | 0.001500 | 3.49 | 33.15 | 1 | 0 |  |
| *Parus major* | 480 | 516 | 0.000561 | 0.000569 | 3.49 | 33.6 | 1 | 0 |  |
| *Parus major* | 40 | 47 | 0.007962 | 0.008000 | 24.89 | M13, 33.15 | 1 | 0 |  |
| *Parus montanus* | 111 | 112 | 0.012474 | . | 0.89 | (GTG)5 | 0 | 0 |  |
| *Passer domesticus* | 18 | 19 | 0.000000 | 0.000000 | 5.26 | 33.6 | 1 | 0 |  |
| *Passer domesticus* | 10 | 11 | 0.003472 | 0.003500 | 9.09 | 33.6, 33.15 | 1 | 0 |  |
| *Passer domesticus* | 139 | 171 | 0.004988 | 0.013500 | 7.02 | (GGAT)4 | 0 | 0 |  |
| *Passer domesticus* | 109 | 136 | 0.008319 | 0.008000 | 19.85 | M13, 33.6 | 1 | 0 |  |
| *Passer domesticus* | 369 | 420 | 0.000631 | 0.001300 | 12.14 | 36.6 | 1 | 0 |  |
| *Passer domesticus* | 96 | 123 | 0.008067 | . | 19.51 | per | 1 | 0 |  |
| *Passerculus sandwichensis* | 116 | 92 | . | 0.024375 | 33.70 | 33.15 | 1 | 0 |  |
| *Passerina cyanea* | 41 | 63 | 0.010407 | 0.011000 | 34.92 | 33.15, 33.6, M13 | 1 | 0 |  |
| *Perisoreus infaustus* | 20 | 20 | 0.000000 | 0.000000 | 0.00 | 33.15 | 1 | 1 | [7] |
| *Petroica australis* | 29 | 29 | . | 0.000772 | 0.00 | 33.15, 33.6 | 1 | 0 |  |
| *Petroica australis* | 32 | 32 | 0.003378 | 0.003273 | 0.00 | 33.15, 33.6 | 1 | 0 |  |
| *Petronia petronia* | 123 | 181 | . | 0.014000 | 32.04 | 33.15 | 1 | 1 | [8] |
| *Phainopepla nitens* | 34 | 48 | 0.006348 | 0.004494 | 0.00 | 33.15 | 1 | 0 |  |
| *Phalacrocorax aristotelis* | 23 | 28 | 0.005411 | . | 17.86 | 33.15 | 1 | 0 |  |
| *Phalaropus lobatus* | 140 | 232 | 0.012301 | . | 1.72 | 33.15, M13 | 1 | 0 |  |
| *Phalaropus tricolor* | 43 | 51 | 0.018015 | 0.021000 | 0.00 | 33.15 | 1 | 0 |  |
| *Phoebastria irrorata* | 12 | 16 | 0.005274 | . | 25.00 | 33.15 | 1 | 0 |  |
| *Phoebastria irrorata* | 128 | 154 | 0.002587 | . | 16.88 | 33.15 | 1 | 0 |  |
| *Phylloscopus trochilus* | 73 | 109 | 0.011974 | . | 33.03 | per | 1 | 0 |  |
| *Pica pica* | 17 | 17 | 0.022409 | . | 3.13 | 33.15 | 1 | 0 |  |
| *Picoides borealis* | 79 | 80 | 0.000000 | 0.000000 | 1.25 | per | 1 | 0 |  |
| *Porphyrio hochstetteri* | 27 | 27 | 0.000000 | 0.000000 | 0.00 | 33.6, pV47-2 | 1 | 1 | [9] |
| *Porphyrio porphyrio* | 74 | 74 | . | 0.001380 | 0.00 | pV47-2, 3'HVR, per | 1 | 0 |  |
| *Progne subis* | 116 | 138 | . | 0.013529 | 18.84 | 33.6 | 1 | 0 |  |
| *Progne subis* | 34 | 52 | 0.004029 | . | 34.62 | 33.6 | 1 | 0 |  |
| *Prunella collaris* | 65 | 110 | 0.005514 | 0.005525 | 0.00 | 33.15, 33.6 | 1 | 0 |  |
| *Prunella modularis* | 132 | 133 | 0.001306 | 0.000100 | 0.80 | 33.15 | 1 | 0 |  |
| *Psaltriparus minimus* | 50 | 50 | . | 0.002000 | 0.00 | per, 33.15, 33.6 | 1 | 0 |  |
| *Puffinus tenuirostris* | 7 | 22 | 0.000000 | 0.000000 | 10.80 | 33.6 | 1 | 0 |  |
| *Pygoscelis adeliae* | 20 | 22 | 0.019444 | 0.013900 | 9.09 | 33.15 | 1 | 1 | [10] |
| *Pygoscelis antarctica* | 76 | 76 | 0.007448 | 0.007400 | 0.00 | (GGAT)4 | 0 | 0 |  |
| *Remiz pendulinus* | 115 | 201 | 0.050125 | . | 6.97 | (GATA)4 | 0 | 0 |  |
| *Riparia riparia* | 136 | 167 | 0.005882 | . | 14.37 | 33.6 | 1 | 0 |  |
| *Riparia riparia* | 131 | 190 | 0.026285 | . | 18.95 | per | 1 | 0 |  |
| *Sayornis phoebe* | 67 | 76 | 0.003980 | . | 11.84 | 33.15, per | 1 | 0 |  |
| *Sericornis frontinalis* | 120 | 137 | 0.002222 | 0.005600 | 12.41 | per, 33.15 | 1 | 0 |  |
| *Serinus canaria* | 45 | 45 | 0.001844 | 0.002000 | 0.00 | 33.15 | 1 | 0 |  |
| *Serinus serinus* | 61 | 61 | . | 0.001429 | 0.00 | (GATA)4 | 0 | 1 | [11] |
| *Serinus serinus* | 124 | 139 | 0.006144 | . | 9.40 | (GATA)4 | 0 | 0 |  |
| *Setophaga ruticilla* | 62 | 108 | 0.005237 | . | 39.81 | (GGAT)4 | 0 | 0 |  |
| *Sialia mexicanus* | 127 | 207 | . | 0.012000 | 18.36 | 33.15, 33.6 | 1 | 0 |  |
| *Sialia sialis* | 21 | 83 | 0.009079 | . | 8.43 | 33.15 | 1 | 0 |  |
| *Spheniscus humboldti* | 49 | 49 | 0.005607 | 0.014500 | 0.00 | 33.15, 33.6 | 1 | 0 |  |
| *Sterna hirundo* | 24 | 29 | 0.014228 | 0.012700 | 0.00 | 33.15 | 1 | 0 |  |
| *Sturnus unicolor* | 270 | 334 | 0.005210 | 0.005000 | 15.87 | 33.15 | 1 | 0 |  |
| *Sturnus vulgaris* | 51 | 62 | 0.003103 | . | 9.68 | 33.6 | 1 | 0 |  |
| *Sturnus vulgaris* | 51 | 62 | 0.004442 | . | 9.68 | 33.15 | 1 | 0 |  |
| *Sturnus vulgaris* | 84 | 92 | 0.002976 | . | 8.70 | 33.15 | 1 | 0 |  |
| *Tachycineta bicolor* | 53 | 86 | 0.006717 | . | 38.37 | per | 1 | 0 |  |
| *Tachycineta bicolor* | 36 | 95 | 0.004495 | . | 62.11 | 33.15, per | 1 | 0 |  |
| *Tachycineta bicolor* | 28 | 111 | 0.045714 | . | 68.47 | per, 33.15 | 1 | 0 |  |
| *Taeniopygia guttata* | 80 | 92 | 0.003289 | . | 2.17 | 33.6, 33.15 | 1 | 0 |  |
| *Tetrao tetrix* | 62 | 66 | 0.008161 | . | 0.00 | 33.6 | 1 | 1 | [12] |
| *Thalassoica antarctica* | 38 | 41 | 0.009368 | . | 7.32 | per | 1 | 0 |  |
| *Thryothorus ludovicianus* | 84 | 84 | 0.017902 | 0.019000 | 0.00 | 33.15 | 1 | 0 |  |
| *Tockus monteiri* | 135 | 135 | 0.014262 | 0.014000 | 0.00 | 33.15 | 1 | 0 |  |
| *Tribonyx mortierii* | 26 | 26 | . | 0.018000 | 0.00 | per | 1 | 0 |  |
| *Troglodytes aedon* | 742 | 790 | 0.001366 | 0.001400 | 8.35 | M13, per | 1 | 0 |  |
| *Turdoides squamiceps* | 186 | 186 | 0.000671 | 0.000700 | 0.00 | 33.15, 33.6 | 1 | 0 |  |
| *Turdus grayi* | 21 | 37 | 0.009099 | . | 37.84 | 33.15 | 1 | 0 |  |
| *Tyrannus tyrannus* | 37 | 64 | 0.027027 | 0.030000 | 42.19 | 33.15 | 1 | 1 | [13] |
| *Tyto alba* | 121 | 122 | 0.004934 | . | 0.82 | (GGAT)4 | 0 | 1 | [14] |
| *Tyto alba* | 89 | 89 | 0.000000 | . | 0.00 | (GGAT)4 | 0 | 1 |  |
| *Upupa epops* | 99 | 108 | 0.022389 | . | 8.33 | 33.15 | 1 | 0 |  |
| *Uria aalge* | 71 | 77 | . | 0.019000 | 7.79 | per | 1 | 1 | [15] |
| *Vireo solitarius* | 36 | 37 | 0.000000 | . | 2.70 | 33.15 | 1 | 0 |  |
| *Wilsonia citrina* | 261 | 356 | . | 0.002400 | 26.69 | 33.15 | 1 | 0 |  |
| *Wilsonia citrina* | 55 | 78 | 0.015810 | . | 29.49 | 33.15, 33.6 | 1 | 0 |  |
| *Zonotrichia albicollis* | 71 | 89 | . | 0.013000 | 17.98 | per, M13 | 1 | 0 |  |
| *Zosterops lateralis* | 54 | 122 | 0.000000 | 0.000000 | 0.00 | 33.6 | 1 | 0 |  |
