# Supplementary material for: Sexual selection does not influence minisatellite mutation rate
Source: BMC Evol Biol. 2009 Jan 8;9:5. doi: 10.1186/1471-2148-9-5 (PMC2636768; doi:10.1186/1471-2148-9-5)
Supplement: Additional file 3 — Supplementary Table 2. Comparative information on extra-pair paternity, mutation rate, sample size and mean number of bands scored as reported by Amos [1] and according to the publications. [file 1471-2148-9-5-S3.doc]

Supplementary Table 2. Comparative information on extra-pair paternity, mutation rate, sample size and mean number of bands scored as reported by Amos [1] and according to the publications.

| Species | Extra-pair paternity in Amos [1] | Extra-pair paternity in publication | Mutation rate in Amos [1] | Mutation rate in publication | Sample size in Amos [1] | Sample size in publication | Mean no. bands in Amos [1] | Mean no. bands in publications |
| --- | --- | --- | --- | --- | --- | --- | --- | --- |
| *Acrocephalus melanopogon* | 27 | 27.27 | 0.0155 | . | . | 18 | . | 17.9 |
| *Actitis hypoleucos* | 15.7 | 15.66 | 0.0071 | 0.006 | 57 | 60 | 47.1 | 47.1 |
| *Alectura lathami* | 27.7 | 27.69 | 0.004 | 0.003333 | 47 | 50 | 26.3 | 26.2 |
| *Anthus spinoletta* | 5.2 | 5.23 | 0.0436 | . | 71 | 681 | 48.8 | 27.2 |
| *Aphelocoma coerulescens* | 1.4 | 0.00 | 0.0055 | 0.005 | 139 | 139 | 40.5 | 40.5 |
| *Athene noctua* | 0 | 0.00 | 0.0036 | 0.0035 | 53 | 53 | 20.95 | 20.95 |
| *Calidris mauri* | 7 | 5.10 | 0.011 | . | 57 | 57 | 32 | 32 |
| *Calonectris diomedea* | 9 | 0.00 | 0.0077 | 0.007 | 34 | 34 | 26.7 | 26.7 |
| *Carduelis tristis* | 14.3 | 14.29 | 0.013 | . | 54 | 54 | 20 | 20 |
| *Carpodacus mexicanus* | 14.4 | 8.40 | 0.0178 | . | 5 | 73 | 22.5 | 22.5 |
| *Charadrius alexandrinus* | 3.9 | 1.31 | 0.0188 | 0.015 | 168 | 167 | 18 | 18 |
| *Charadrius hiaticula* | 0 | 0.00 | 0.0168 | . | 50 | 50 | 17.9 | 17.9 |
| *Charadrius semipalmatus* | 4.7 | 4.71 | 0.0012 | 0.0012 | 62 | 62 | 27.3 | 27.3 |
| *Corvus monedula* | 0 | 0.00 | 0.0044 | 0.0043859 | . | 74 | . | 15.41 |
| *Cyanoliseus patagonus* | 1.2 | 0.00 | 0.0033 | 0.003 | 165 | 164 | 25.6 | 25.6 |
| *Euplectes orix* | 14.1 | 17.59 | 0.0188 | 0.019 | 145 | 145 | 10.26 | 10.6 |
| *Falco naumanni* | 3.4 | 3.45 | 0.0033 | 0.0041 | 84 | 67 | 10.9 | 10.9 |
| *Falco sparverius* | 11.2 | 11.24 | 0.0259 | . | 79 | 79 | 12.2 | 12.2 |
| *Fregata minor* | 2.2 | 1.09 | 0.0165 | 0.0164 | 90 | 91 | 14.1 | 14.1 |
| *Grallina cyanoleuca* | 2.9 | 2.91 | 0.0023 | . | 100 | 100 | 21.3 | 11.3 |
| *Grallina cyanoleuca* | 2.9 | 2.91 | 0.0023 | . | 100 | 44 | 21.3 | 11.3 |
| *Lanius minor* | 0 | 0.00 | 0.0049 | . | 136 | 136 | 21 | 21 |
| *Manorina melanophrys* | 4.2 | 4.20 | 0.0768 | 0.003333 | 24 | 23 | 30.4 | 33.3 |
| *Miliaria calandra* | 5.3 | 4.55 | 0.0083 | 0.0082644 | 36 | 36 | 13.4 | 13.4 |
| *Oceanites oceanicus* | 0 | 0.00 | 0.005 | 0.005 | 126 | 63 | 38.1 | 38.1 |
| *Oceanodroma leucorhoa* | 0 | 0.00 | 0.0078 | 0.008 | 42 | 42 | 21.4 | 21.4 |
| *Otus asio* | 0 | 0.00 | 0.0072 | . | 76 | 76 | 51 | 51.4 |
| *Otus flammeolus* | 0 | 0.00 | 0.0031 | 0.0031 | 37 | 37 | 44 | 44 |
| *Panurus biarmicus* | 14.4 | 14.44 | 0.0041 | . | 187 | 148 | 26 | 26 |
| *Parus ater* | 25.3 | 25.32 | 0.0085 | 0.0063 | 118 | 118 | 14.03 | 14.03 |
| *Parus atricapillus* | 17 | 16.98 | 0.0941 | . | 53 | 44 | 20.26 | 20.26 |
| *Parus montanus* | 1 | 0.89 | 0.012 | . | 111 | 111 | 19.5 | 19.5 |
| *Phainopepla nitens* | 0 | 0.00 | 0.0063 | 0.004494 | 34 | 34 | 27.8 | 27.8 |
| *Phalaropus lobatus* | 1.7 | 1.72 | 0.012 | . | . | 140 | . | 51.1 |
| *Phoebastria irrorata* | 25 | 25.00 | 0.004 | . | . | 12 | . | 15.8 |
| *Phoebastria irrorata* | 25 | 16.88 | 0.004 | . | . | 128 | . | 15.1 |
| *Pygoscelis antarctica* | 0 | 0.00 | 0.0074 | 0.0074 | 76 | 76 | 10.6 | 10.6 |
| *Remiz pendulinus* | 6.9 | 6.97 | 0.0568 | . | 105 | 119 | 11.4 | 11.4 |
| *Serinus canaria* | 0 | 0.00 | 0.0019 | 0.002 | . | 45 | . | 24.1 |
| *Serinus serinus* | 9.4 | 9.40 | 0.0044 | . | 153 | 124 | 21 | 21 |
| *Setophaga ruticilla* | 40 | 39.81 | 0.0052 | . | 62 | 62 | 15.4 | 15.4 |
| *Sterna hirundo* | 0 | 0.00 | 0.0118 | 0.0127 | 29 | 24 | 20.5 | 20.5 |
| *Sturnus unicolor* | 16 | 15.87 | 0.0052 | 0.005 | 270 | 270 | 17.06 | 17.06 |
| *Thalassoica antarctica* | 9.5 | 7.32 | 0.0094 | . | 38 | 38 | 30.9 | 30.9 |
| *Thryothorus ludovicianus* | 0 | 0.00 | 0.0197 | 0.019 | 84 | 84 | 13.3 | 13.3 |
| *Tockus monteiri* | 0 | 0.00 | 0.0143 | 0.014 | 135 | 135 | 16.62 | 16.62 |
| *Vireo olivaceus* | 57.9 | 57.89 | 0 | 0 | . | 1 | . | 15.1 |
| *Zonotrichia albicollis* | 31.8 | 17.98 | 0.013 | 0.013 | . | 71 | . | . |
| *Zosterops lateralis* | 0 | 0.00 | 0 | 0 | . | 54 | . | 13.7 |
