# Supplementary figures and images for: Sexual selection does not influence minisatellite mutation rate
Source: BMC Evol Biol. 2009 Jan 8;9:5. doi: 10.1186/1471-2148-9-5 (PMC2636768; doi:10.1186/1471-2148-9-5)

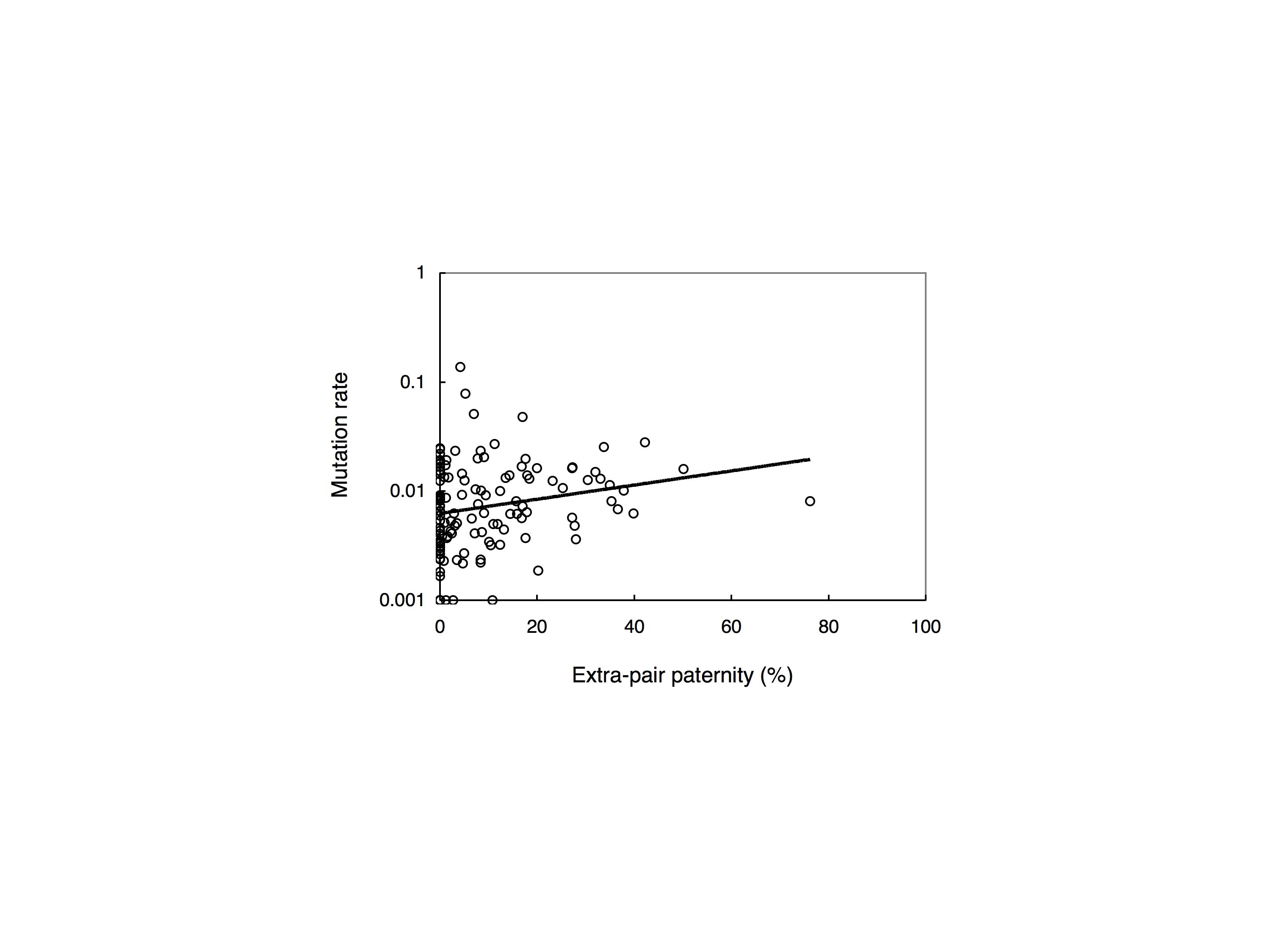

Supplement: Additional file 4 — Response figure 1. Positive relationship between mutation rate and extra-pair paternity (% extra-pair young) in different species of birds. Mutation rate was log10-transformed with a constant of 0.001 being added to avoid values of zero. The line is the linear regression line. [file 1471-2148-9-5-S4.doc]
