# Supplementary material for: Sexual selection does not influence minisatellite mutation rate
Source: BMC Evol Biol. 2009 Jan 8;9:5. doi: 10.1186/1471-2148-9-5 (PMC2636768; doi:10.1186/1471-2148-9-5)
Supplement: Supplementary file 5 [file 1471-2148-9-5-S5.doc]

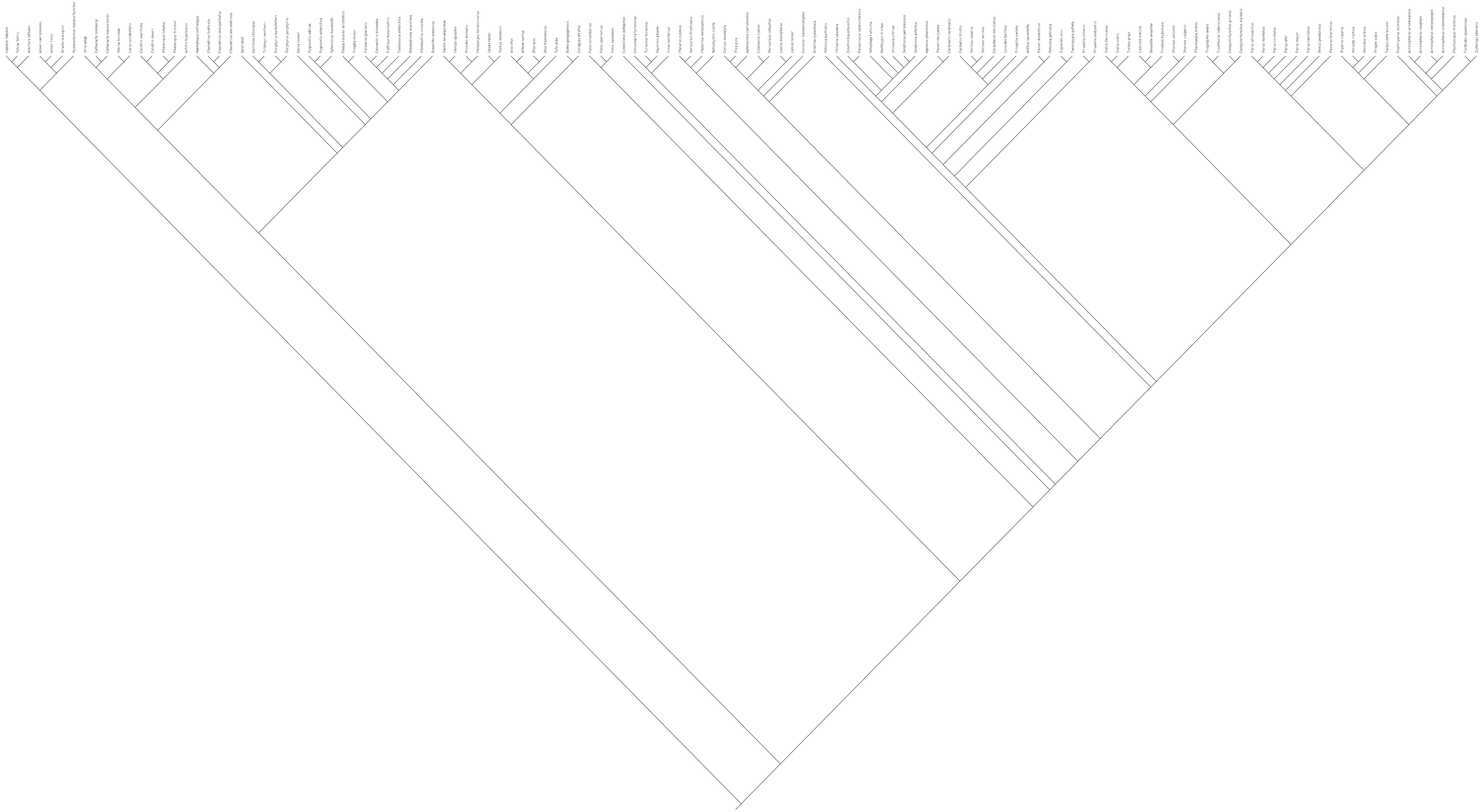
Lagopus lagopus
Tetrao tetrix
Alectura lathami
Anser caerulescens
Anser rossi
Branta leucopsis
Hymenolaimus malacorhynchos
Uria aalge
Catharacta lonnbergi
Catharacta maccormicki
Sterna hirundo
Larus occidentalis
Calidris maritima
Calidris mauri
Phalaropus lobatus
Phalaropus tricolor
Actitis hypoleucos
Haematopus ostralegus
Charadrius hiaticula
Charadrius semipalmatus
Charadrius alexandrinus
Apus apus
Gallinula chloropus
Tribonyx mortieri
Porphyrio hochstetteri
Porphyrio porphyrio
Gavia immer
Pygoscelis adeliae
Pygoscelis antarctica
Spheniscus humboldti
Phalacrocorax aristotelis
Fregata minor
Fulmarus glacialis
Calonectris diomedea
Puffinus tenuirostris
Thalassoica antarctica
Oceanodroma leucorhoa
Phoebastria irrorata
Oceanites oceanicus
Dacelo novaeguineae
Merops apiaster
Picoides borealis
Melanerpes formicivorus
Upupa epops
Tockus monteiri
Asio otus
Athene noctua
Otus asio
Otus flammeolus
Tyto alba
Buteo galapagoensis
Coragyps atratus
Falco columbarius
Falco sparverius
Falco naumanni
Cyanoliseus patagonus
Cercomacra tyrannina
Tyrannus tyrannus
Sayornis phoebe
Vireo solitarius
Malurus cyaneus
Sericornis frontinalis
Manorina melanophrys
Notiomystis cincta
Corvus monedula
Pica pica
Aphelocoma coerulescens
Cyanopica cyanus
Perisoreus infaustus
Lanius bucephalus
Lanius minor
Corcorax melanoramphos
Grallina cyanoleuca
Petroica australis
Miliaria calandra
Zonotrichia albicollis
Passerculus sandwichensis
Setophaga ruticilla
Geothlypis trichas
Wilsonia citrina
Dendroica caerulescens
Dendroica petechia
Agelaius phoeniceus
Passerina cyanea
Cardinalis cardinalis
Carduelis tristis
Serinus canaria
Serinus serinus
Carpodacus mexicanus
Loxoides bailleui
Fringilla coelebs
Anthus spinoletta
Passer domesticus
Petronia petronia
Euplectes orix
Taeniopygia guttata
Prunella collaris
Prunella modularis
Sialia mexicanus
Sialia sialis
Turdus grayi
Luscinia svecica
Oenanthe oenanthe
Ficedula hypoleuca
Sturnus unicolor
Sturnus vulgaris
Phainopepla nitens
Troglodytes aedon
Tryothorus ludovicianus
Campylorhynchus griseus
Campylorhynchus nuchalis
Parus atricapillus
Parus montanus
Parus cristatus
Parus ater
Parus major
Parus caeruleus
Remiz pendulinus
Panurus biarmicus
Riparia riparia
Hirundo rustica
Delichon urbica
Progne subis
Tachycineta bicolor
Psaltriparus minimus
Acrocephalus arundinaceus
Acrocephalus vaughani
Acrocephalus melanopogon
Acrocephalus schoenobaenus
Phylloscopus trochilus
Turdoides squamiceps
Zosterops lateralis
